# New Cytotoxic Anthraquinone Derivatives from a Deep-Sea-Derived *Aspergillus* sp. SCSIO 41331

**DOI:** 10.3390/md24060214

**Published:** 2026-06-15

**Authors:** Ziyi Wu, Zehan Zheng, Weimao Zhong, Qianting Jiang, Mengjing Cong, Haozhe Zhang, Fazuo Wang, Yonghong Liu, Hailiang Hu, Junfeng Wang

**Affiliations:** 1 State Key Laboratory of Tropical Oceanography, Guangdong Key Laboratory of Marine Materia Medica, South China Sea Institute of Oceanology, Chinese Academy of Sciences, Guangzhou 510301, China; wuziyi24@mails.ucas.ac.cn (Z.W.); weimaozhong@scsio.ac.cn (W.Z.); jiangqianting25@mails.ucas.ac.cn (Q.J.); c3021632921@163.com (M.C.); wangfazuo@scsio.ac.cn (F.W.); yonghongliu@scsio.ac.cn (Y.L.); 2 University of Chinese Academy of Sciences, 19 Yuquan Road, Beijing 100049, China; 3 Department of Biochemistry, SUSTech Homeostatic Medicine Institute, School of Medicine, Southern University of Science and Technology, Shenzhen 518000, China; 12531553@mail.sustech.edu.cn (Z.Z.); 12331441@mail.sustech.edu.cn (H.Z.); 4 Sanya Institute of Ocean Eco-Environmental Engineering, Sanya 572000, China

**Keywords:** anthraquinone, diphenyl ether, deep-sea-derived fungi, anticancer activity

## Abstract

Two new anthraquinone derivatives, (±)-1′-*O*-methyl-6-chloroaverantin (**1a** and **1b**) and 6-chloroaverythrin (**2**), and one new diphenyl ether 1-((*E*)-but-2-en-2-yl)-3,8-dihydroxy-6-((*E*)-4-hydroxybut-2-en-2-yl)-4,9-dimethyl-11*H*-dibenzo[b,e][1,4]dioxepin-11-one (**3**), along with six known compounds, were isolated from the fungus *Aspergillus* sp. SCSIO 41331 collected from the deep-sea sediment in the cold-seep area of the South China Sea. Elucidation of planar structures was achieved via 1D and 2D NMR and mass spectrometry, whereas stereochemistry was validated through optical rotation and NOE correlations, chiral phase HPLC analysis and NMR calculation. All compounds were assessed for antitumor activity, among which compound **4** displayed moderate antiproliferative activity against HT29 cells and suppressed colony expansion.

## 1. Introduction

The deep sea, where oceanic water depths exceed 1000 m, occupies 95% of the ocean’s volume and represents the largest and most unexplored biome of Earth’s biosphere [1,2]. Deep-sea fungi exhibit remarkable adaptability and metabolic activity under extreme environmental conditions such as high hydrostatic pressure, low temperature, high salinity, and lack of light and oxygen, thereby yielding a wealth of marine natural products, which play a crucial role in biotechnology, species communication adaptation, and pharmaceutical applications [2,3,4]. Marine cold seeps, as typical chemolithotrophic ecosystems, are characterized by methane-rich fluid emissions and unique sulfur redox reactions [5,6]. Therefore, deep-sea cold-seep-derived fungi have developed a unique metabolic mechanism and physiological process during their evolution, representing one of the most promising new sources for producing important diverse or untapped medicinal lead compounds [4,7,8,9,10]. Moreover, secondary metabolites isolated from cold-seep environments in recent years have demonstrated significant biological activity. Among many other classes of natural products, anthraquinones are one class possessing broad bioactivity, exhibiting antibacterial [11], anti-inflammatory [12], and antitumor properties [13].

**Figure 1:**
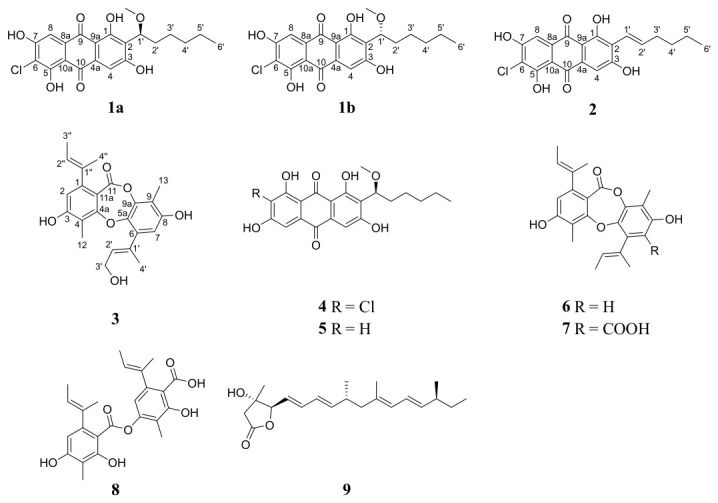
Structures of compounds **1**–**9**.

Therefore, to uncover more novel bioactive secondary metabolites from deep-sea cold-seep-derived fungi, we have pursued continuous chemical investigation. As a result, a fungal strain of *Aspergillus* sp. collected from deep-sea sediment in the cold-seep area of the South China Sea caught our attention, from which we isolated and identified two unprecedented anthraquinones, (±)-1′-*O*-methyl-6-chloroaverantin (**1a** and **1b**) and 6-chloroaverythrin (**2**), one new diphenyl ether derivative, 1-((*E*)-but-2-en-2-yl)-3,8-dihydroxy-6-((*E*)-4-hydroxybut-2-en-2-yl)-4,9-dimethyl-11*H*-dibenzo[b,e][1,4]dioxepin-11-one (**3**), along with six known compounds, 1′-*O*-methyl-7-chloroaverantin (**4**) [14], 1′-*O*-methylaverantin (**5**) [14], folipastatin (**6**) [15], 7-carboxyfolipastatin (**7**) [16], agonodepside B (**8**) [17], and trichonafurin A (**9**) [18] (Figure 1). This paper performs the isolation and structural elucidation of new compounds **1**–**3**, along with the biological evaluation of the isolated compounds.

## 2. Results and Discussion

Compound **1** was obtained as an orange powder. The molecular formula of **1** was determined to be C_21_H_21_ClO_7_ due to the HRESIMS ion peaks at *m*/*z* 419.0921 [M − H]^−^ (calc. for C_21_H_20_ClO_7_, 419.0903), indicating 11 degrees of unsaturation. The UV spectrum of **1** exhibited a characteristic absorption peak of the anthraquinone moiety at λ_max_ of 290 nm, which was beneficial for UV-guided separation. The ^1^H NMR (Table 1) and HSQC spectra of **1** showed 15 sp^3^-hybridized protons and 2 sp^2^-hybridized protons. The ^13^C NMR spectrum indicated the presence of 21 carbons, namely, 2 carbonyls (*δ*_C_ 183.2, C-9/10), 10 aromatic quaternary carbons, 3 methines carbons (including 1 oxygen-bearing carbon at *δ*_C_ 75.1 and 2 aromatic carbons at 115.9 and 109.0), 4 methylene carbons, 1 methoxy carbon, and 1 methyl carbon (*δ*_C_ 14.1, C-6′).

**Figure 2:**
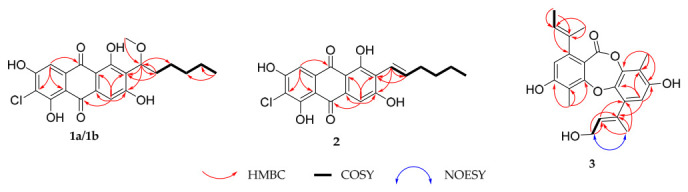
Key HMBC, COSY and NOESY correlations of compounds **1**−**3**.

The UV−Vis and ^1^H and ^13^C NMR data of **1** were compared with those of the known compound 1′-*O*-methyl-7-chloroaverantin (**4**) isolated from *Aspergillus* sp., revealing highly similar data and an identical molecular formula. The primary distinction comprised the change of the chemical shifts of the ^13^C NMR signal at the C-8 (from *δ*_C_ 108.3 in **4** to *δ*_C_ 115.9 in **1**) and of the adjacent C-10a and C-8a signals, indicating a change in the substituent position on the benzene ring. The position of H-8 on the benzene ring in **1** was confirmed by the key HMBC correlations between H-8 and C-10a, C-6, and C-9. Moreover, the ^13^C NMR signal of C-9 shifted downfield to the same chemical shift as that of C-10, indicating that C-9 and C-10 underwent nearly identical deshielding effects from adjacent substituents. Moreover, the 1,5-dihydroxy anthraquinone moiety exhibited a nearly symmetrical structure, resulting in chemical shifts of the ^13^C NMR signals at positions C-10a and C-8a that were opposite to those observed in the known compound **4**. Additionally, the HMBC correlations of H-4/C-2/C-3/C-4a/C-9a/C-10; H-1′/C-2 and H-2′/C-2 determined the connection between C-1′ and C-2 in the side chain and the positions of the remaining carbons on the benzene ring (Figure 2). Concurrently, the HMBC correlation between the oxygen methyl proton attached to C-1′ confirmed the presence of the oxygen methyl group. Therefore, compound **1** was described as 1′-*O*-methyl-6-chloroaverantin.

Due to the baseline ECD curve and the barely measurable specific rotation value, compound **1** was presumed to be a racemate. Subsequently, **1** was separated using a chiral column, yielding (+)-**1** and (−)-**1**, respectively (Figure S8). Furthermore, the dichroism calculations (CD) spectrum of the absolute configuration at the C-1′ stereogenic center of compound **1a**, as well as its negative optical rotation values (${\left[\alpha \right]}_{D}^{25}$ −52.5 (*c* 0.24, MeOH)), were consistent with those of (1′*S*)-1′-*O*-Methyl-7-chloroaverantin (compound **3** in [14]), thereby confirming that compound **1a** possessed the same *S*-configuration, and compound **1b** possessed the *R*-configuration. Moreover, through the time-dependent density functional theory (TDDFT) calculations at the B3LYP/6-311G(d,p) level in Gaussian 09, the average calculated specific optical rotation values obtained for (1′*R*)-**1** and (1′*S*)-**1** were +345.3 and −376.0, respectively, which matched the experimental values of (+)-**1b** (${\left[\alpha \right]}_{D}^{25}$ +60.0 (*c* 0.23, MeOH)) and (−)-**1a** (${\left[\alpha \right]}_{D}^{25}$ −52.5 (*c* 0.24, MeOH)). Therefore, the absolute configurations of (+)-**1b** and (−)-**1a** were (1′*R*)-**1** and (1′*S*)-**1**, respectively.

Compound **2** was obtained as a purple powder. The molecular formula of **2** was determined to be C_20_H_17_ClO_6_ due to the HRESIMS ion peaks at *m*/*z* 387.0649 [M − H]^−^ (calc. for C_20_H_16_ClO_6_, 387.0641). The structures of compounds **2** and **1** were highly similar based on the ^1^H and ^13^C NMR spectroscopic data. By comparing the ^1^H and ^13^C NMR data of **1** and **2**, it was found that the main difference was the variation in side-chain structure. The C-1′ at *δ*_C_ 119.3 and C-2′ at *δ*_C_ 136.4 were connected by a double bond to form two sp^2^-methine groups in **2**, whereas C-1′ was connected to one methoxy group and one sp^3^-methylene group in **1**. The COSY correlations from H-1′ to H-2′; H-2′/H-3′; H-3′/H-4′; H-4′/H-5′ and H-5′/H-6′ and the HMBC correlations of H-1′ with C-2 confirmed the position of the side chain and the correlation between C-1′ and C-2. Additionally, the H-1′ signal at 6.61 and the H-2′ signal at 6.88 exhibited a large coupling constant (16.0 Hz), suggesting that H-1′/H-2′ adopt a *trans*-configuration. Furthermore, in the HMBC spectrum, the correlations of H-8/C-10a/C-6/C-9 and H-4/C-2/C-3/C-4a/C-9a/C-10 confirmed that compound **2** possessed the same anthraquinone structure as that of compound **1**. Therefore, compound **2** was established as 6-chloroaverythrin.

Compound **3** was obtained as a light yellow powder. The molecular formula of **3** was determined to be C_23_H_24_O_6_ due to the HRESIMS ion peaks at *m*/*z* 395.1500 [M − H]^−^ (calc. for C_23_H_23_O_6_, 395.1500), indicating 12 degrees of unsaturation. Since deviations were observed in the chemical shift signals of H-2 and some hydrogen atoms in the high-field region when analyzing the 2D-NMR spectrum, it was inferred that the DMSO-*d*_6_ solvent effect might be responsible. Subsequently, acetone-*d*_6_ was used as the solvent to eliminate the solvent effect. The ^1^H NMR spectrum recorded in acetone-*d*_6_ exhibited two aromatic protons at *δ*_H_ 6.63 (s, H-2) and 6.53 (s, H-7); five methyl protons at *δ*_H_ 2.17 (s, H-12), 2.19 (s, H-13), 2.08 (d, *J* = 1.2 Hz, H-4′), 1.67 (dd, *J* = 6.7, 1.3 Hz, H-3″) and 1.87 (t, *J* = 1.3 Hz, H-4″); one methylene proton at *δ*_H_ 4.31(d, *J* = 6.2 Hz, H-3′); and two olefinic methine protons at *δ*_H_ 5.64 (td, *J* = 6.0, 1.5 Hz, H-2′) and 5.38 (m, H-2″). The ^13^C NMR spectrum indicated the presence of 23 carbons, namely, 1 carbonyl, 12 quaternary carbons (including 10 aromatic quaternary carbons), 4 methine carbons, 1 hydroxymethyl group carbon, and 5 methyl carbons.

Based on the ^1^H and ^13^C NMR data, compound **3** was essentially identical to compound **6**, indicating that compound **3** was a depsidone, and they shared the same skeletal structure. Their notable distinction was the alteration of the chemical shifts at the C-3′ (from *δ*_C_ 14.1 in **6** to *δ*_C_ 59.4 in **3**) and the distinct deshielded shifted resonance of C-2′ (from *δ*_C_ 125.1 in **6** to *δ*_C_ 132.4 in **3**). Specifically, this referred to the replacement of the methyl group at the C-3′ position with a hydroxymethyl group. Further HMBC correlations from the methyl proton at C-12 to C-3/C-4/C-4a and the hydroxymethyl group at C-3′ to C-1′/C-2′ confirmed the presence of the methyl and hydroxymethyl group.

Since the positions at C-1 and C-2 in compound **3** can easily be interchanged, while still satisfying the observed HMBC correlation, in order to further confirm the relative configuration of compound **3**, the gauge invariant atomic orbital (GIAO) method at the B3LYP/6-31G(d,p)/PCM(acetone-*d*_6_) was adopted to calculate the ^13^C NMR data of the two possible isomers of compound **3** (Tables S1 and S2). The experimental ^13^C NMR data of **3** match well with those for the isomer **3a** (DP4+ probability: 100%) (Figures S27 and S28). Thus, the planar structure of compound **3** was determined. Furthermore, the relative configuration of compound **3** was determined by NOESY experiments and NMR data. The NOESY spectrum revealed the correlations from H_2_-3′ to H_3_-4′, indicating that the double bond between H-3′ and H-4′ adopted the *E*-configuration. Moreover, the chemical shifts of C-4″ at *δ*_C_ 17.7 and C-4′ at *δ*_C_ 18.4 were consistent with the ^13^C NMR data of the known compounds 7-chlorofolipastatin [15] and folipastatin [15], confirming the *E*-configuration of the double bond at H-3″/H-4″. Therefore, compound **3** was established as 1-((*E*)-but-2-en-2-yl)-3,8-dihydroxy-6-((*E*)-4-hydroxybut-2-en-2-yl)-4,9-dimethyl-11*H*-dibenzo[b,e][1,4]dioxepin-11-one.

Anthraquinones have a parent structure consisting of three fused rings and two carbonyl groups which are typically substituted with hydroxyl, hydroxymethyl, methyl, methoxy, and carboxyl groups, and they exhibit a wide range of pharmacological activities, such as anti-neurodegenerative and anti-hepatopathic effects, anticancer and anti-inflammatory activities, etc. [19]. Because chloride/chlorine is the dominant element in the ocean, there are far more chlorinated compounds than brominated compounds [20]. Exogenous addition of NaBr is usually required to produce bromoanthraquinone, whereas the addition of NaI does not yield iodoanthraquinone [21]. Currently, most anthraquinone compounds are halogen-substituted at the C-7 position (compounds **4** and **5**). In contrast, compounds **1** and **2** are chlorinated at the C-6 position, indicating that C-6 substitution in the anthraquinone skeleton represents a structurally novel feature.

**Figure 3:**
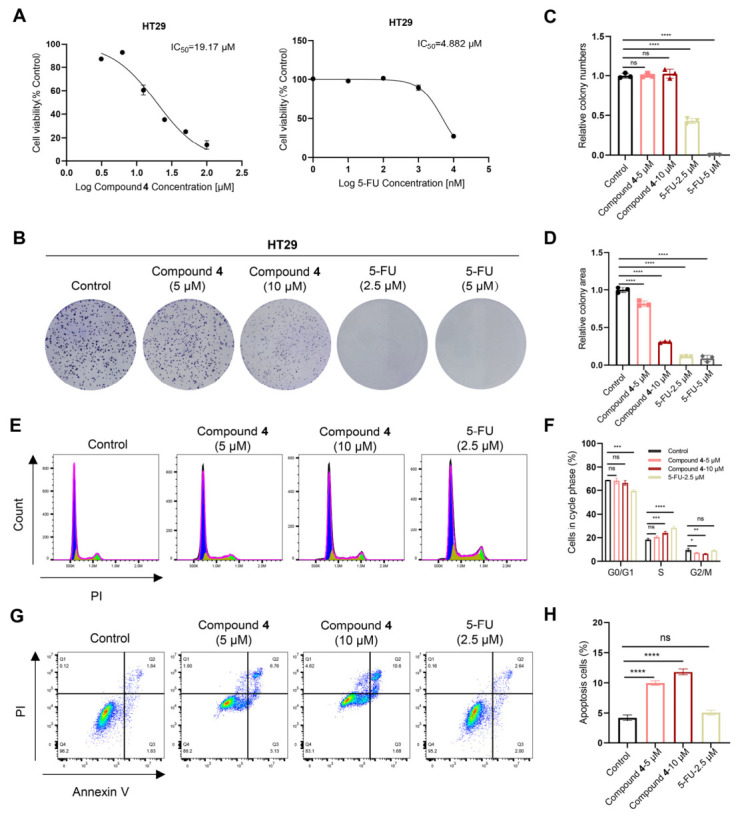
Compound **4** suppresses colony expansion and induces apoptosis-associated cell death in HT29 cells. (**A**) Dose–response curves of **4** and 5-FU in HT29 cells. Cell viability was measured after treatment with increasing concentrations of **4** or 5-FU, and IC_50_ values were calculated. (**B**) Representative images of colony formation assays in HT29 cells treated with **4** or 5-FU. (**C**) Quantification of colony number. (**D**) Quantification of colony area. (**E**) Representative PI-stained cell-cycle histograms after treatment with **4** or 5-FU. (**F**) Quantification of the percentage of cells in G0/G1, S, and G2/M phases. (**G**) Representative annexin V/PI flow cytometry plots. (**H**) Quantification of total apoptotic cells. Total apoptotic cells were defined as the sum of early and late apoptotic populations. Data are presented as mean ± SD (*n* = 3). Statistical significance was determined by one-way ANOVA, followed by Dunnett’s multiple comparisons test. ns, not significant; *, *p* < 0.05; **, *p* < 0.01; ***, *p* < 0.001; ****, *p* < 0.0001.

All isolated compounds were evaluated for antitumor activity against a panel of human cancer cell lines. Among them, all compounds exhibited IC_50_ values above 50 µM, except for compound **4**, which displayed moderate inhibitory activity against colon cancer cells, with an IC_50_ value of 19.17 µM (Figure 3A). As a preliminary evaluation of cytotoxicity in non-tumor-derived cells, compound **4** was tested in 293T cells and showed an IC_50_ value of 27.73 μM, while the positive control 5-FU exhibited a high level of cytotoxicity with an IC_50_ value of 5.84 μM (Figure S29). To evaluate the long-term antiproliferative effect of **4**, colony formation assays were performed in HT29 cells. Compound **4** at 5 and 10 μM significantly reduced relative colony area without markedly affecting colony number, indicating impaired colony expansion (Figure 3B–D). As expected, the positive control 5-FU strongly suppressed colony formation, reducing both colony number and colony area (Figure 3B–D). To explore the potential biological basis underlying the reduced colony expansion, cell-cycle distribution and apoptosis were further examined. Cell-cycle analysis showed that compound **4** caused a modest alteration in cell-cycle distribution, mainly characterized by a slight increase in the S-phase population at 10 μM. In contrast, 5-FU induced a more pronounced S-phase accumulation (Figure 3E,F). Annexin V/PI staining showed that compound **4** significantly increased the proportion of apoptotic cells in HT29 cells in a concentration-dependent manner (Figure 3G,H). Compared with the control group, compound **4** at 5 and 10 μM significantly increased total apoptotic cells in HT29 cells in a concentration-dependent manner, whereas 5-FU at 2.5 μM showed only a modest effect in this assay (Figure 3G,H). Collectively, these results indicate that compound **4** exhibits moderate antiproliferative activity against HT29 cells and suppresses colony expansion. The reduced colony growth induced by compound **4** may be mainly linked to apoptosis-associated cell death, while cell-cycle alterations appear to play a limited or minor role.

## 3. Materials and Methods

### 3.1. General Experimental Procedures

Optical rotations were obtained using an Anton MCP-500 polarimeter (Anton Paar, Austria). HRESIMS data were recorded on a Bruker maXis Q-TOF mass spectrometer (Bruker, Fallanden, Switzerland). NMR spectra were recorded using a Bruker AV 500 MHz or AVANCE iii HD 700 MHz NMR spectrometer (Bruker, Falländen, Switzerland) with TMS as the internal standard. Chemical shifts were denoted by *δ* values in the ^1^H and ^13^C spectra, with the coupling constant denoted by *J* values. The UV and CD spectra were recorded on a Shimadzu UV-2600 PC spectrometer (Shimadzu, Kyoto, Japan) and a Chirascan circular dichroism spectrometer (Applied Photophysics, Surrey, UK), respectively. HPLC was undertaken on Agilent 1260 and Hitachi Primaide devices equipped with YMC ODS SERIES (YMC-Pack ODS-A, YMC Co., Ltd. (Kyoto, Japan)., 250 × 10 mm I.D., S-5 μm, 12 nm), a NanoChrom column (ChromCore 120 C18, NanoChrom Technologies Co., Ltd. (Suzhou, China), 250 × 10 mm I.D., S-5 μm), and a chiral column (CHIRALPAK IC, DAICEL (Shanghai, China), 4.6 mm Ø × 250 mmL, S-5 μm). Column chromatography (CC) was performed on silica gel (200–300 mesh, Jiangyou Silica Gel Development Co., Yantai, China), Sephadex LH-20 (40–70 μm, Amersham Pharmacia Biotech AB (Sweden, Uppsala) and Combi Flash (Irregular C18, 40–63 μm, Santai Tech (Changzhou, China). The TLC plates with silica gel GF254 (0.4–0.5 mm, Qingdao Marine Chemical Factory, Qingdao, China) were applied for analysis and preparative TLC.

### 3.2. Fungal Material

The fungal strain *Aspergillus* sp. was isolated from a cold-seep-sediment fungus, which was collected from the South China Sea (depth 1138 m) in July 2020. It was identified by Associate Professor Xiao-Yong Zhang, College of Marine Science, South China Agricultural University, as *Aspergillus* sp. and named as *Aspergillus* sp. SCSIO 41331 (accession no. LC105691). A voucher specimen has been preserved in the Laboratory of Tropical Marine Bio-Resources and Ecology, South China Sea Institute of Oceanology, Chinese Academy of Sciences, Guangzhou, China.

### 3.3. Extraction and Isolation

The *Aspergillus* sp. strain was inoculated onto agar plates and incubated at 25 °C for 7 days. The seed culture was prepared by inoculating spores of strain SCSIO 41331 into three 500 mL flasks, each containing 150 mL of seed medium (malt extract: 15 g, sea salt: 2.5 g, distilled water: 1 L), and incubated at 25 °C on a 180 r min^−1^ rotating shaker for 3 days. The seed culture was then transferred into 1 L × 60 conical flasks with solid rice medium (each flask contained 150 g of rice, 3 g of sea salt, and 150 mL of naturally sourced water), and the large-scale fermentation of the strain was carried out at 25 °C for 30 days. The total rice culture was crushed and extracted with EtOAc five times to yield 71.5 g of crude gum.

### 3.4. Purification

The crude material (71.5 g) was separated by silica gel column chromatography (CC) using petroleum ether (PE), ethyl acetate (EA), and methanol mixtures (90:10:0–0:0:100, *v*/*v*) and divided into nine fractions (Fr.1–9), based on TLC characteristics. Fr.3 was similarly separated on the Sephadex LH-20 column eluting with H_2_O/MeOH (0–100%), yielding nine subfractions (Fr.3-1–3-9). Fr.3-8 was purified by semi-preparative HPLC eluting with 86% MeOH/H_2_O with 0.04% FA to obtain **2** (2.29 mg, t_R_ 49.6 min). Fr.3–4 was further separated into seven subfractions (Fr.3-4-1–3-4-7) through Sephadex LH-20 column eluting with H_2_O/MeOH (0–100%). Fr.3-4-5 was further purified by semi-preparative HPLC eluting with 59% MeOH/H_2_O to obtain **3** (1.43 mg, t_R_ 25.4 min). Fr.3–5 was purified by semi-preparative HPLC eluting with 68% MeOH/H_2_O with 0.04% FA to obtain **6** (21.66 mg, t_R_ 27.7 min) and **7** (6.2 mg, t_R_ 36.8 min). Fr.3–7 was purified by semi-preparative HPLC eluting with 70% MeCN/H_2_O with 0.04% FA to obtain **9** (1.58 mg, t_R_ 28.5 min). Fr.4 was further separated into six subfractions (Fr.4-1−4-6) through Sephadex LH-20 column eluting with H_2_O/MeOH (0–100%). Fr.4-4 underwent semi-preparative HPLC purification using an ODS column to obtain three subfractions (Fr.4-4-1–Fr.4-4-3), eluting with 75% MeCN/H_2_O with 0.04% formic acid (FA). Fr.4-4-1 was further purified by semi-preparative HPLC eluting with 86% MeOH/ H_2_O with 0.04% FA to obtain **1** (1.82 mg, t_R_ 32.3 min) and another subfraction (Fr.4-4–1-1). Compound **1** was further separated into **1a** (0.24 mg, t_R_ 51.3 min) and **1b** (0.23 mg, t_R_ 46.8 min) by semi-preparative chiral column eluting with 85% MeOH/H_2_O with 0.04% FA. Fr.4–5 was purified by semi-preparative HPLC eluting with 91% MeOH/H_2_O with 0.04% FA to obtain **4** (18.62 mg, t_R_ 16.7 min). Fr.4-4-2 was purified by semi-preparative HPLC eluting with 90% MeOH/ H_2_O with 0.04% FA to obtain **5** (1.89 mg, t_R_ 16.4 min). Fr.4–3 was purified by semi-preparative HPLC eluting with 75% MeOH/H_2_O with 0.04% FA to obtain **8** (2.49 mg, t_R_ 31.7 min).

1′-*O*-methyl-6-chloroaverantin (±**1**): yellow powder; (−)-**1a** (${\left[\alpha \right]}_{D}^{25}$ −52.5 (*c* 0.24, MeOH)); (+)-**1b** (${\left[\alpha \right]}_{D}^{25}$ +60.0 (*c* 0.23, MeOH)); **1a** (UV (MeOH) λ_max_ (log ε)): 215 (3.15), 222 (3.17), 242 (2.80), 263 (2.89), 276 (2.76), 316 (3.08) nm; **1b** (UV (MeOH) λ_max_ (log ε)): 217 (3.02), 220 (3.02), 242 (2.63), 263 (2.73), 280 (2.48), 316 (2.30) nm; **1a** (CD (MeOH) Δε (nm)): 209 (0.42), 225 (−1.20), 270 (−0.18), 294 (−0.46), 334 (0.03), and 388 (−0.49) nm; **1b** (CD (MeOH) Δε (nm)): 204 (−0.20), 223 (0.77), 233 (0.29), 238 (0.37), 268 (−0.18), 312 (0.06), 341 (−0.19), and 374 (0.17) nm; ^1^H NMR and ^13^C NMR data, Table 1; HR-ESI-MS *m*/*z* 419.0921 [M − H]^−^ (calc. for C_21_H_20_ClO_7_, 419.0903).

6-chloroaverythrin (**2**): yellow powder; ^1^H NMR and ^13^C NMR data, Table 1; HR-ESI-MS *m*/*z* 387.0649 [M − H]^−^ (calc. for C_20_H_16_ClO_6_, 387.0641), 775.1381 [2M − H]^−^ (calc. for C_40_H_33_Cl_2_O_12_, 775.1355).

1-((*E*)-but-2-en-2-yl)-3,8-dihydroxy-6-((*E*)-4-hydroxybut-2-en-2-yl)-4,9-dimethyl-11*H*-dibenzo[b,e][1,4]dioxepin-11-one (**3**): yellow powder; ^1^H NMR and ^13^C NMR data, Table 2; HR-ESI-MS *m*/*z* 395.1500 [M − H]^−^ (calc. for C_23_H_25_O_6_, 395.1500),791.3077 [2M − H]^−^ (calc. for C_46_H_47_O_12_, 791.3073).

### 3.5. Calculation Details

Quantum chemical calculations were implemented via density functional theory (DFT) using the Gaussian 09 software package [22]. A preliminary conformation was identified using Spartan 14 software (Wavefunction Inc., Irvine, CA, USA) coupled with the MMFF force field to detect low-energy conformers. On this basis, the stable conformers obtained from using the MMFF force field were first subjected to geometric optimization at the B3LYP/6-31G(d) level, and the solvation effects of methanol were incorporated using the SMD continuum model. Ultimately, electronic excitation energies and rotational strengths were obtained through time-dependent density functional theory (TDDFT) calculations at the B3LYP/6-311G(d,p) level, which enabled the calculation of specific optical rotation (OR) value. The NMR chemical shifts were calculated via the gauge invariant atomic orbital (GIAO) method [23] at the B3LYP/6-31G(d,p) level with PCM (acetone-*d*_6_), accounting for solvent effects to reproduce the experimental solvent environment. Furthermore, the computed NMR shifts for the **3a** and **3b** isomers were subjected to DP4+ probability analysis using a publicly available Excel-based tool (https://sarotti-NMR.weebly.com).

### 3.6. Cell Culture

The human cancer cell lines, HT29 and the 293T cells, were purchased from Procell Life Science & Technology Co., Ltd. (Wuhan, China). Cells were cultured in the recommended culture medium supplemented with 10% fetal bovine serum and 1% penicillin–streptomycin at 37 °C in a humidified atmosphere containing 5% CO_2_.

### 3.7. Cytotoxicity Assay

Colon cancer cell viability was assessed using a CellTiter-Lumi^TM^ Luminescent Cell Viability Assay (Beyotime, Shanghai, China), according to the manufacturer’s instructions [24]. Briefly, cells were seeded into 96-well plates at an appropriate density and allowed to attach overnight. Cells were then treated with increasing concentrations of compound **4** or the positive control 5-FU for 48 h. The luminescent signal was measured using a Synergy H1 microplate reader (BioTek, Shanghai, China). Cell viability was normalized to the untreated control group and expressed as a percentage. Dose–response curves were generated using nonlinear regression analysis, and IC_50_ values were calculated using GraphPad Prism 9.

### 3.8. Colony Formation Assay

For 2-D clonogenic assay, cells were then seeded in 6-well plates at a concentration of 1000 cells per well. The cells were incubated at 37 °C and allowed to adhere overnight. Cells were then treated with compound **4** (5 and 10 μM) or the positive control 5-FU (2.5 and 5 μM) and cultured for 5–7 days. At the end of the incubation period, the 6-well plates were washed with 1 × PBS, and the cells were fixed by adding 100% methanol and incubating them at 4 °C for 10 min. The cells were then stained with 0.5% crystal violet for 10 min at room temperature before being washed twice in double-distilled (dd) H_2_O. Colonies were imaged, and quantitative analysis of colony number and colony area was performed using ImageJ software version 1.51j8 and GraphPad Prism 9.

### 3.9. Cell-Cycle Analysis

For cell-cycle analysis, HT29 cells were seeded in 6-well plates and allowed to attach overnight. Cells were then treated with compound **4** (5 and 10 μM) or 5-FU (2.5 μM) for 48 h. After treatment, both floating and adherent cells were collected, washed twice with cold PBS, and stained using a Cell Cycle and Apoptosis Analysis Kit (Servicebio, Wuhan, China), according to the manufacturer’s instructions. Briefly, cells were fixed with 70% ethanol at 4 °C overnight, followed by incubation with RNase A and propidium iodide (PI) staining solution for 30 min at room temperature in the dark. DNA content was detected using a NovoCyte 2000R flow cytometer (Beijing, China), and cell-cycle distribution was analyzed using FlowJo software version 10.8.1. The percentages of cells in G0/G1, S, and G2/M phases were quantified based on DNA content.

### 3.10. Apoptosis Analysis

For apoptosis analysis, cell apoptosis was assessed using an Annexin V-FITC/PI Apoptosis Detection Kit (YEASEN, Shanghai, China), according to the manufacturer’s instructions. Briefly, HT29 cells were seeded in 6-well plates and treated with compound **4** (5 and 10 μM) or 5-FU (2.5 μM) for 48 h. After treatment, both floating and adherent cells were harvested, washed twice with cold PBS, and resuspended in binding buffer. Cells were then stained with annexin V-FITC and PI for 15 min at room temperature in the dark. Apoptotic cells were detected using a NovoCyte 2000R flow cytometer, and data were analyzed using FlowJo software version 10.8.1. Early apoptotic cells were defined as annexin V-FITC-positive and PI-negative cells, whereas late apoptotic cells were defined as annexin V-FITC-positive and PI-positive cells. Total apoptotic cells were calculated as the sum of early and late apoptotic populations.

## 4. Conclusions

In summary, two novel anthraquinones and one new diphenyl ether derivative were isolated and identified from the fermentation extract of the fungus *Aspergillus* sp., which originated from a deep-sea sediment. Notably, a pair of enantiomers was isolated from compound **1,** separated using a chiral column, yielding (+)-**1b** and (−)-**1a**, respectively. Additionally, the planar structures of the new compounds were determined by 1D and 2D NMR spectroscopy, HRESIMS, while their relative and absolute configurations were established based on optical rotation data, NOESY, and ^13^C NMR. All compounds were assessed for their antitumor activity. Among them, compound **4** displayed moderate antiproliferative activity against HT29 cells and suppressed colony expansion. Preliminary structure–activity relationship analysis indicated that the halogen substitution site influenced the cytotoxicity of anthraquinone analogues. Compared to compound **4** and other C-7-chloro analogues with cytotoxic activity [14], compounds **1** and **2**, which were C-6-chloro analogues, exhibited a significant decrease in antitumor activity. This indicated that the halogen substitution site was critical to the antitumor activity of anthraquinones.

## Figures and Tables

**Table 1:** ^1^H and ^13^C NMR data for **1**, **2** and **4** (*δ* in ppm, DMSO-*d*_6_).

Pos.	1 ^a^		2 ^a^		4 ^b^	
	*δ*_C_, Type	*δ*_H_ Mult. (*J* in Hz)	*δ*_C_, Type	*δ*_H_ Mult. (*J* in Hz)	*δ*_C_, Type	*δ*_H_ Mult. (*J* in Hz)
1	162.2, C		160.2, C		162.2, C	
2	119.2, C		116.4, C		119.3, C	
3	162.0, C		160.5, C		163.6, C	
4	107.3, CH	7.08, s	106.9, CH	7.13, s	109.7, C	7.12, s
4a	133.3, C		131.2, C		133.2, C	
5	160.5, C		159.9, C		108.0, CH	7.14, s
6	112.6, C		111.8, C		160.4, C	
7	161.9, C		160.0, C		113.0, C	
8	115.9, CH	6.69, s	115.9, CH	6.65, brs	159.4, C	
8a	131.7, C		130.8, C		108.8, C	
9	183.2, C		182.7, C		188.1, C	
9a	109.0, C		108.2, C		108.5, C	
10	183.2, C		182.7, C		181.0, C	
10a	101.7, C		100.5, C		131.8, C	
1′	75.1, CH	4.78, t (7.2)	119.3, CH	6.61, d (16.0)	74.8, CH	4.78, t (7.1)
2′	32.8, CH_2_	1.96, m 1.83, m	136.4, CH	6.68, dt (16.0, 7.0)	32.6, CH_2_	1.98, m 1.82, m
3′	25.5, CH_2_	1.33–1.24 overlap	33.6, CH_2_	2.21, q (7.2)	25.4, CH_2_	1.33–1.24 overlap
4′	31.3, CH_2_	1.24, m	30.8, CH_2_	1.42, m	31.2, CH_2_	1.24, m
5′	22.2, CH_2_	1.24, m	21.3, CH_2_	1.34, h (7.2)	22.1, CH_2_	1.24, m
6′	14.1, CH_3_	0.83, t (7.0)	13.4, CH_3_	0.90, t (7.3)	14.0, CH_3_	0.84, overlap
1′-OCH_3_	56.4, CH_3_	3.14, s			56.3, CH_3_	3.15, s

^a 1^H and ^13^C NMR measured at 700 and 175 MHz, respectively. ^b 1^H and ^13^C NMR measured at 500 and 125 MHz, respectively.

**Table 2:** ^1^H and ^13^C NMR data for **3** and **6** (*δ* in ppm).

Pos.	3 ^a^		3 ^b^		6 ^c^	
	*δ*_C_, Type	*δ*_H_ Mult. (*J* in Hz)	*δ*_C_, Type	*δ*_H_ Mult. (*J* in Hz)	*δ*_C_, Type	*δ*_H_ Mult. (*J* in Hz)
1	149.1, C		148.5, C		147.7, C	
2	112.8, CH	6.63, s	116.1, CH	5.93, s	112.0, CH	6.57, s
3	160.1, C		162.1, C		159.8, C	
4	114.6, C		113.4, C		113.5, C	
4a	162.8, C		162.1, C		161.7, C	
5a	143.4, C		142.0, C		141.8, C	
6	136.4, C		134.9, C		135.7, C	
7	111.9, CH	6.53, s	110.4, CH	6.36, s	111.1, CH	6.43, s
8	153.3, C		152.0, C		152.6, C	
9	116.1, C		114.5, C		114.6, C	
9a	144.7, C		144.6, C		143.3, C	
11	164.2, C		164.0, C		163.7, C	
11a	112.8, C		-		110.8, C	
12	9.2, CH_3_	2.19, s	9.2, CH_3_	1.87, s	8.4, C	2.05, s
13	9.0, CH_3_	2.17, s	9.3, CH_3_	2.03, s	9.1, C	2.06, s
1′	134.8, C		133.5, C		135.6, C	
2′	132.4, CH	5.64, td (6.0, 1.5)	131.1, CH	5.44, t (6.2)	125.1, CH	5.48, m
3′	59.4, CH_2_	4.31, d (6.2)	58.1, CH_2_	4.10, d (6.1)	14.1, CH_3_	1.73, dd (6.8, 1.4)
4′	18.5, CH_3_	2.08, d (1.2)	18.4, CH_3_	1.90, s	17.4, CH_3_	1.98, s
1″	136.8, C		138.0, C		133.7, C	
2″	124.4, CH	5.38, m	120.2, CH	5.22, q (6.8)	123.7, CH	5.34, m
3″	14.1, CH_3_	1.67, dd (6.7, 1.3)	14.0, CH_3_	1.60, d (6.6)	13.8, CH_3_	1.65, dd (6.8, 1.4)
4″	17.6, CH_3_	1.87, t (1.3)	17.7, CH_3_	1.68, s	17.9, CH_3_	1.78, s

^a^ Recorded in Acetone-*d*_6_, ^1^H, and ^13^C NMR, measured at 700 and 175 MHz, respectively. ^b^ Recorded in DMSO-*d*_6_, ^1^H, and ^13^C NMR, measured at 700 and 175 MHz, respectively. ^c^ Recorded in DMSO-*d*_6_, ^1^H, and ^13^C NMR, measured at 500 and 125 MHz, respectively.

## Data Availability

The original contributions presented in this study are included in the article. Further inquiries can be directed to the corresponding author(s).

## Supplementary Materials

The following supporting information can be downloaded at: https://www.mdpi.com/article/10.3390/md24060214/s1, including ITS sequence data of *Aspergillus* sp. SCSIO 41331, 1D and 2D NMR spectra and HRESIMS data for the novel compounds, and UV and CD spectra of **1**, and structures of the two isomers of **3** used in the DP4+ calculation, and linear regression analysis of calculated ^13^C NMR shifts of **3a** (up) and **3b** (down) against the experimental shifts of **3**, and preliminary cytotoxicity evaluation of compound **4** and 5-FU in 293T cells, and energies of dominative conformers of **3a** and **3b** at B3LYP/6-311G(d,p) (acetone), and DP4+ analysis of calculated ^13^C NMR data of compound **3a** (Isomer1) and **3b** (Isomer2).
