# Supplementary material for: New Cytotoxic Anthraquinone Derivatives from a Deep-Sea-Derived Aspergillus sp. SCSIO 41331
Source: Mar Drugs. 2026 Jun 15;24(6):214. doi: 10.3390/md24060214 (PMC13301339; doi:10.3390/md24060214)
Supplement: Supplementary file 1 [file marinedrugs-24-00214-s001.zip › marinedrugs-4311240-supplementary.pdf]

Supplementary Information

New cytotoxic anthraquinone derivatives from a deep-sea-derived *Aspergillus* sp. SCSIO 41331

Ziyi Wu[1,3,#], Zehan Zheng[2,#], Weimao Zhong[1,3,4], Qianting Jiang[1,3], Mengjing Cong[1,3], Haozhe Zhang[2], Fazuo Wang[1,3,4], Yonghong Liu[1,3,4], Hailiang Hu[2,*], Junfeng Wang[1,3,4,*]

1 State Key Laboratory of Tropical Oceanography, Guangdong Key Laboratory of Marine Materia Medica, South China Sea Institute of Oceanology, Chinese Academy of Sciences, Guangzhou 510301, China;
2 Department of Biochemistry, SUSTech Homeostatic Medicine Institute, School of Medicine, Southern University of Science and Technology, Shenzhen, 518000, China;
3 University of Chinese Academy of Sciences, 19 Yuquan Road, Beijing 100049, China;
4 Sanya Institute of Ocean Eco-Environmental Engineering, Sanya 572000, China

List of supporting information

The ITS gene sequences data for *Aspergillus* sp. SCSIO 41331 3

Figure S1. The ^{1}H NMR spectrum of **1** in DMSO-d_6. 4

Figure S2. The ^{13}C NMR spectrum of **1** in DMSO-d_6 4

Figure S3. The HSQC spectrum of **1** in DMSO-d_6. 5

Figure S4. The ^{1}H-^{1}H COSY spectrum of **1** in DMSO-d_6. 5

Figure S5. The HMBC spectrum of **1** in DMSO-d_6. 6

Figure S6. The UV spectra of **1a** in MeOH 6

Figure S7. The UV spectra of **1b** in MeOH. 7

Figure S8. The CD spectra of **1a** and **1b** in MeOH. 7

Figure S9. The HRESIMS spectrum of **1**. 8

Figure S10. The ^{1}H NMR spectrum of **2** in DMSO-d_6 8

Figure S11. The ^{13}C NMR spectrum of **2** in DMSO-d_6. 9

Figure S12. The HSQC spectrum of **2** in DMSO-d_6. 9

Figure S13. The ^{1}H-^{1}H COSY spectrum of **2** in DMSO-d_6. 10

Figure S14. The HMBC spectrum of **2** in DMSO-d_6. 10

Figure S15. The HRESIMS spectrum of **2**. 11

Figure S16. The ^{1}H NMR spectrum of **3** in DMSO-d_6 11

Figure S17. The ^{13}C NMR spectrum of **3** in DMSO-d_6 12

Figure S18. The HSQC spectrum of **3** in DMSO-d_6. 12

Figure S19. The ^{1}H-^{1}H COSY spectrum of **3** in DMSO-d_6. 13

Figure S20. The NOESY spectrum of **3** in DMSO-d_6. 13

Figure S21. The HMBC spectrum of **3** in DMSO-d_6. 14

Figure S22. The ^{1}H NMR spectrum of **3** in acetone-d_6. 14

Figure S23. The ^{13}C NMR spectrum of **3** in acetone-d_6. 15

Figure S24. The HSQC spectrum of **3** in acetone-d_6 15

Figure S25. The HMBC spectrum of **3** in acetone-d_6. 16

Figure S26. The HRESIMS spectrum of **3**. 16

Figure S27. Structures of the two isomers of **3** used in the DP4+ calculation 17

Figure S28. Linear regression analysis of calculated ^{13}C NMR shifts of **3a** (up) and **3b** (down) against the experimental shifts of **3**......18
Figure S29. Preliminary cytotoxicity evaluation of compound **4** and 5-FU in 293T cells.......19
Table S1. Energies of dominative conformers of **3a** and **3b** at B3LYP/6-311G(d,p) (acetone) 19
Table S2. DP4$^+$ analysis of calculated ^{13}C NMR data of compound **3a** (Isomer1) and **3b** (Isomer2) 21

The ITS gene sequences data for *Aspergillus* sp. SCSIO 41331

TTCCGTAGGGTGAACCTGCGGAAGGATCATTACTGAGTGCGGGCTGCCTCCGGGC
GCCCAACCTCCCACCCGTGAATACCTAACACTGTTGCTTCGGCGGGGAACCCCCT
CGGGGGCGAGCCGCCGGGGACTACTGAACTTCATGCCTGAGAGTGATGCAGTCT
GAGTCTGAATATAAAATCAGTCAAAACTTTCAACAATGGATCTCTTGGTTCCGGC
ATCGATGAAGAACGCAGCGAACTGCGATAAGTAATGTGAATTGCAGAATTCAGT
GAATCATCGAGTCTTTGAACGCACATTGCGCCCCCTGGCATTCCGGGGGGCATGC
CTGTCCGAGCGTCATTGCTGCCCATCAAGCCCGGCTTGTGTGTTGGGTCGTCGTCC
CCCCCGGGGGACGGGCCCGAAAGGCAGCGGCGGCACCGTGTCCGGTCCTCGAGC
GTATGGGGCTTTGTCACCCGCTCGACTAGGGCCGGCCGGGCGCCAGCCGACGTCT
CCAACCATTTTTCTTCAGGTTGACCTCGGATCAGGTAGGGATACCCGCTGAACTT
AAGCATATCAATA

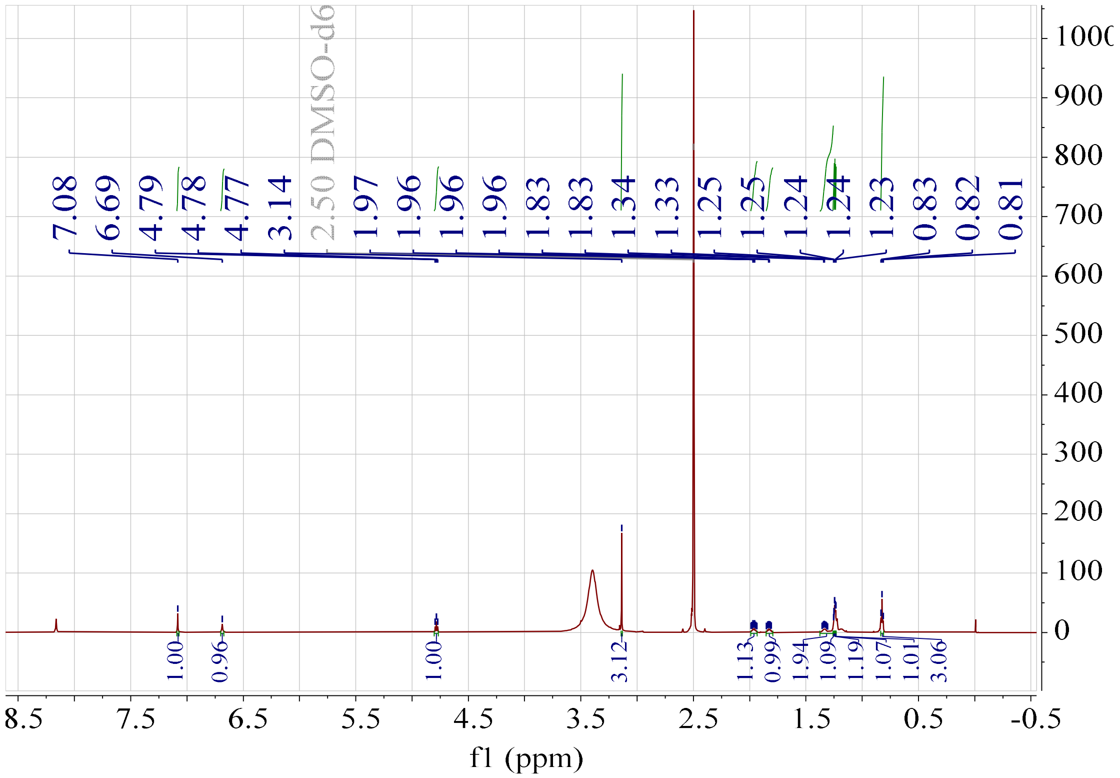

Figure S1. The ^{1}H NMR spectrum of **1** in DMSO-*d*$_6$.

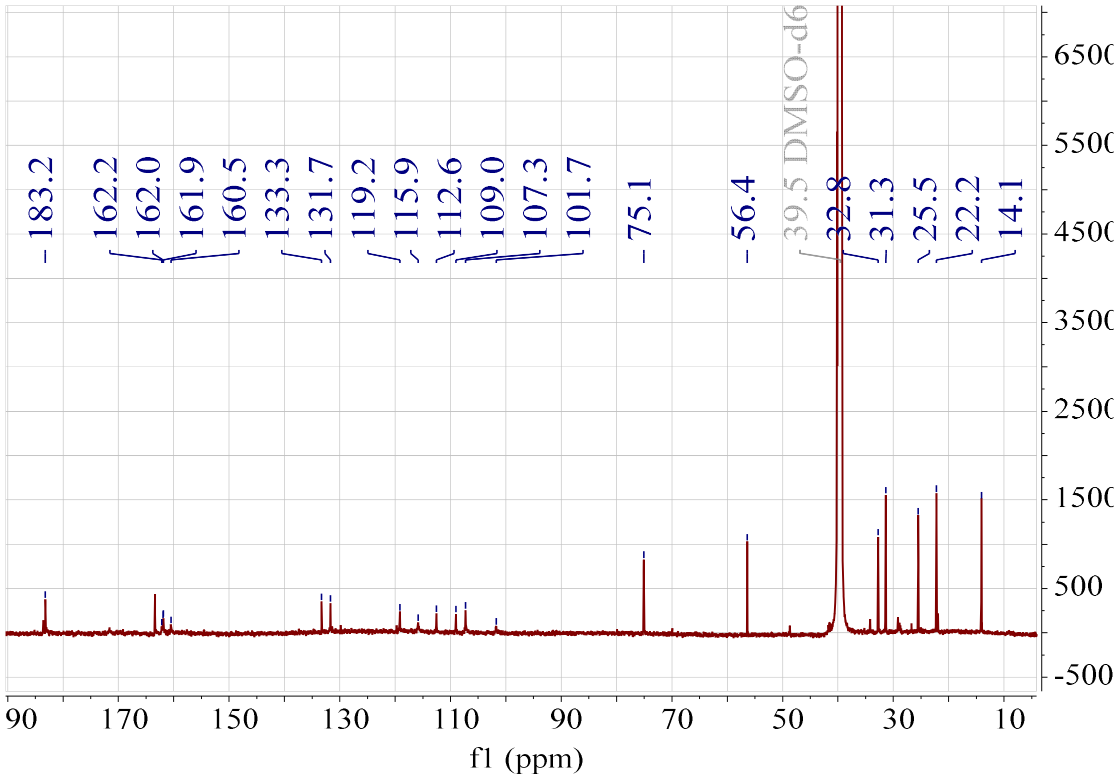

Figure S2. The ^{13}C NMR spectrum of **1** in DMSO-*d*$_6$.

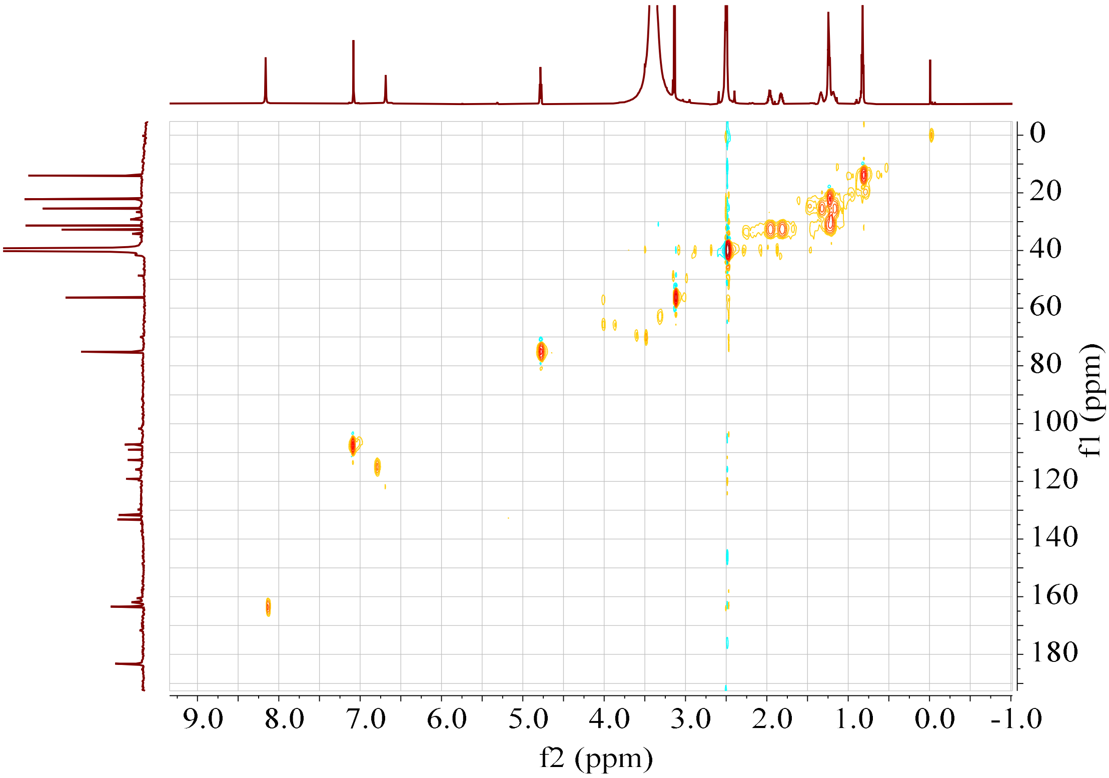

Figure S3. The HSQC spectrum of **1** in DMSO-d_6.

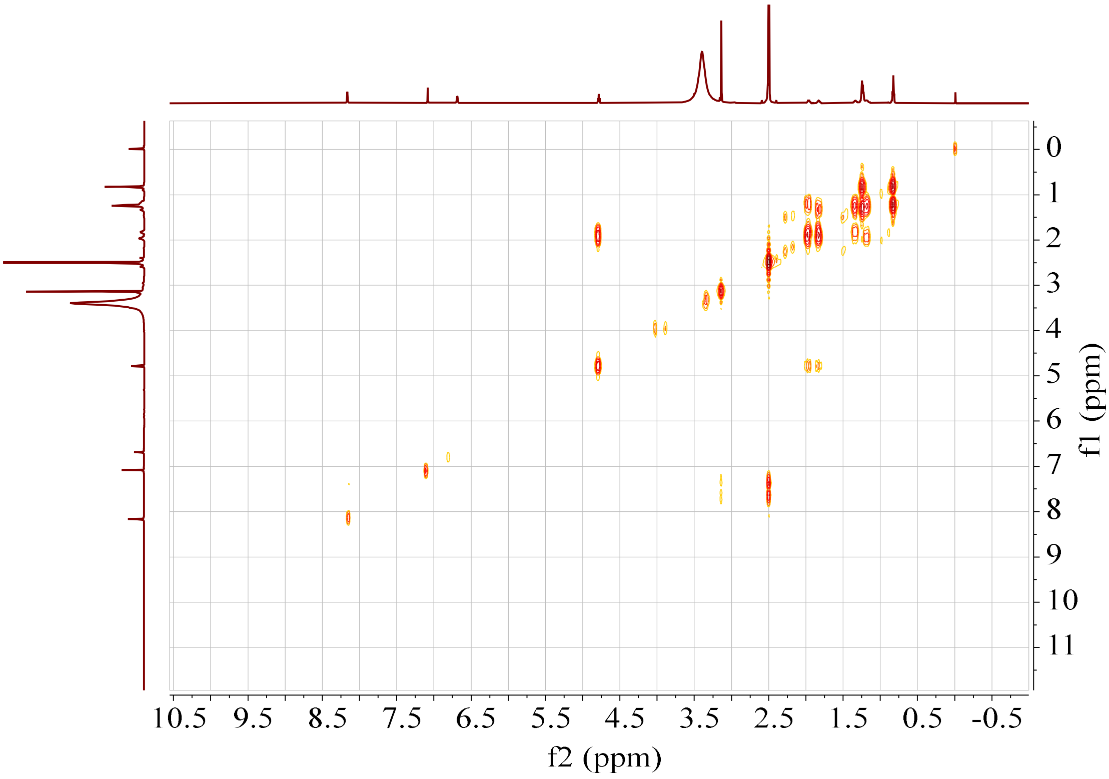

Figure S4. The ^{1}H-^{1}H COSY spectrum of **1** in DMSO-d_6.

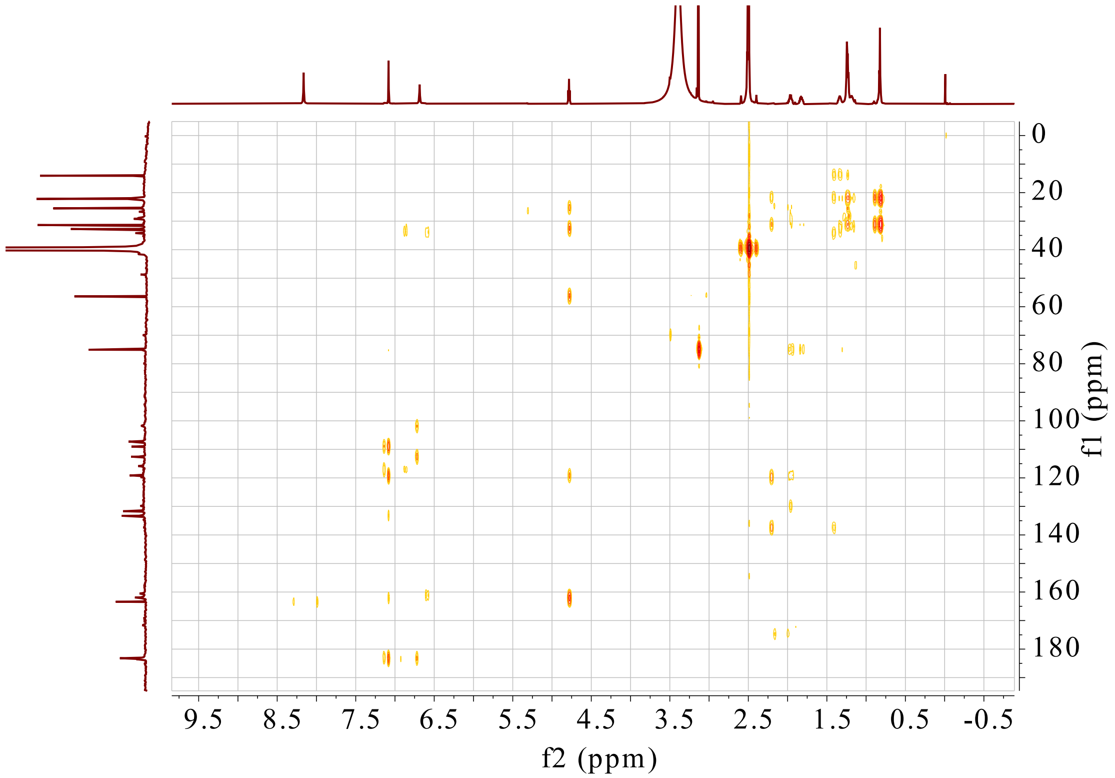

Figure S5. The HMBC spectrum of **1** in DMSO-d_6.

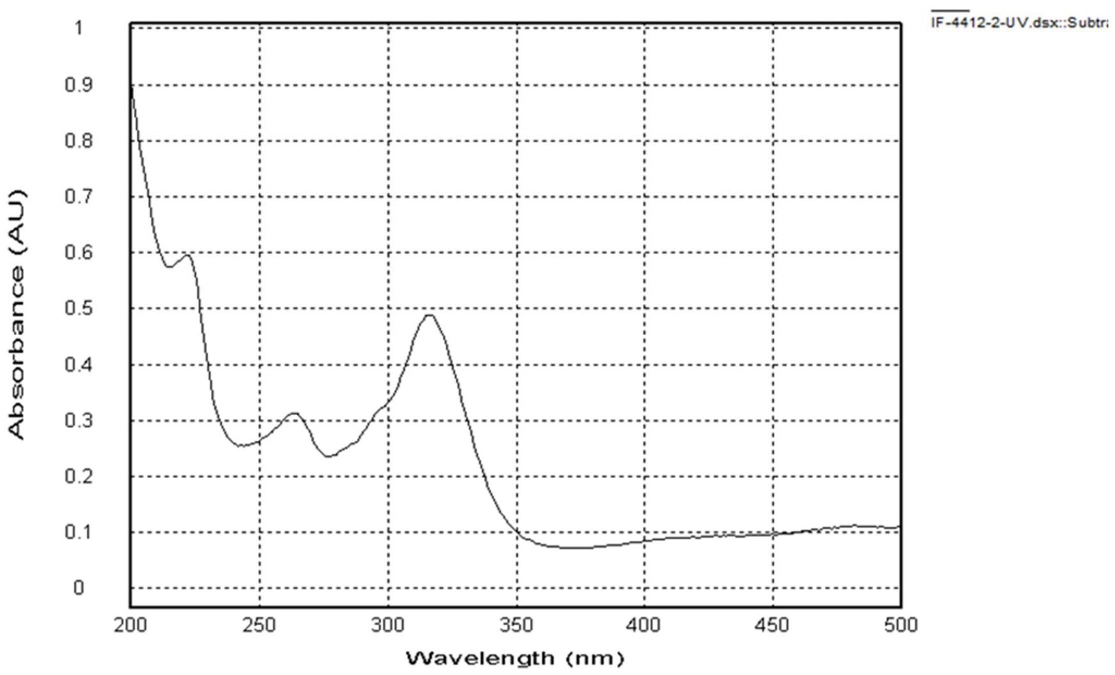

Figure S6. The UV spectra of **1a** in MeOH.

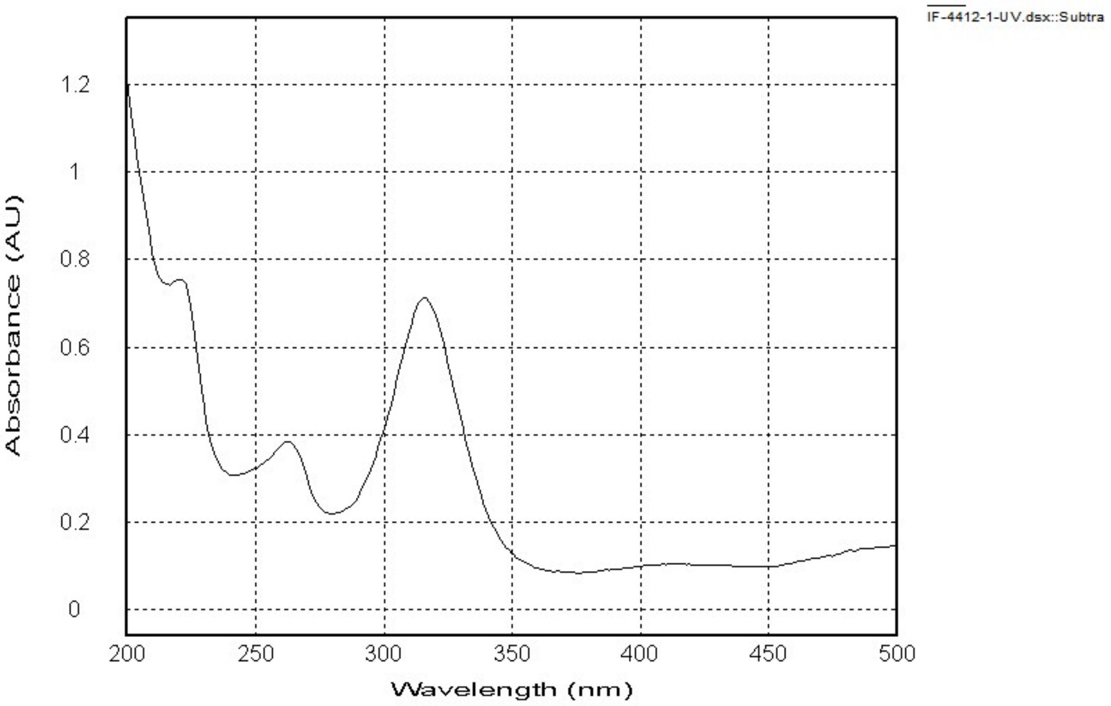

Figure S7. The UV spectra of **1b** in MeOH.

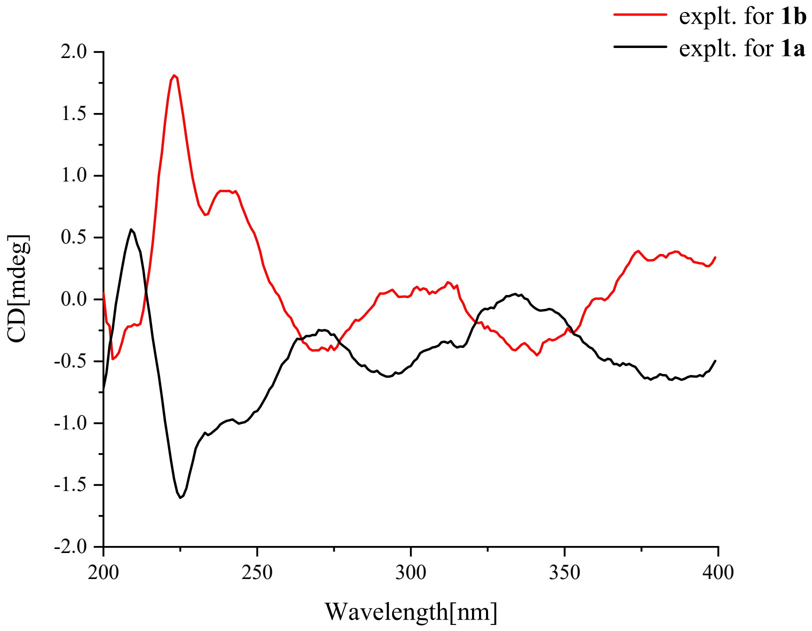

Figure S8. The CD spectra of **1a** and **1b** in MeOH.

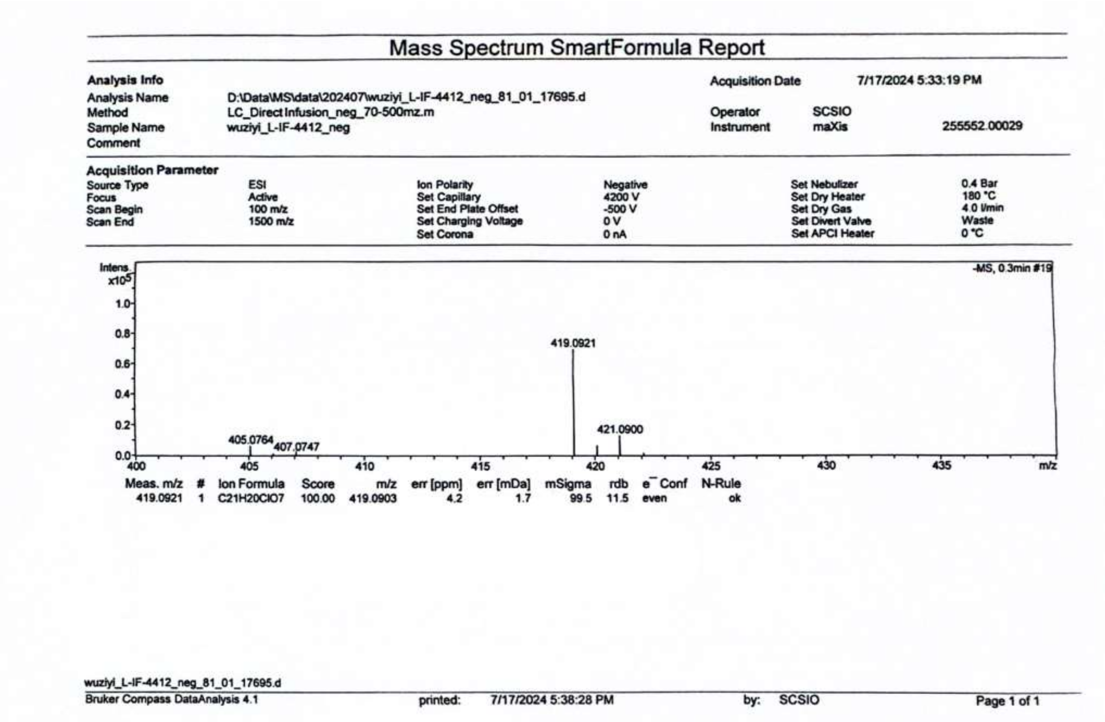

Figure S9. The HRESIMS spectrum of **1**.

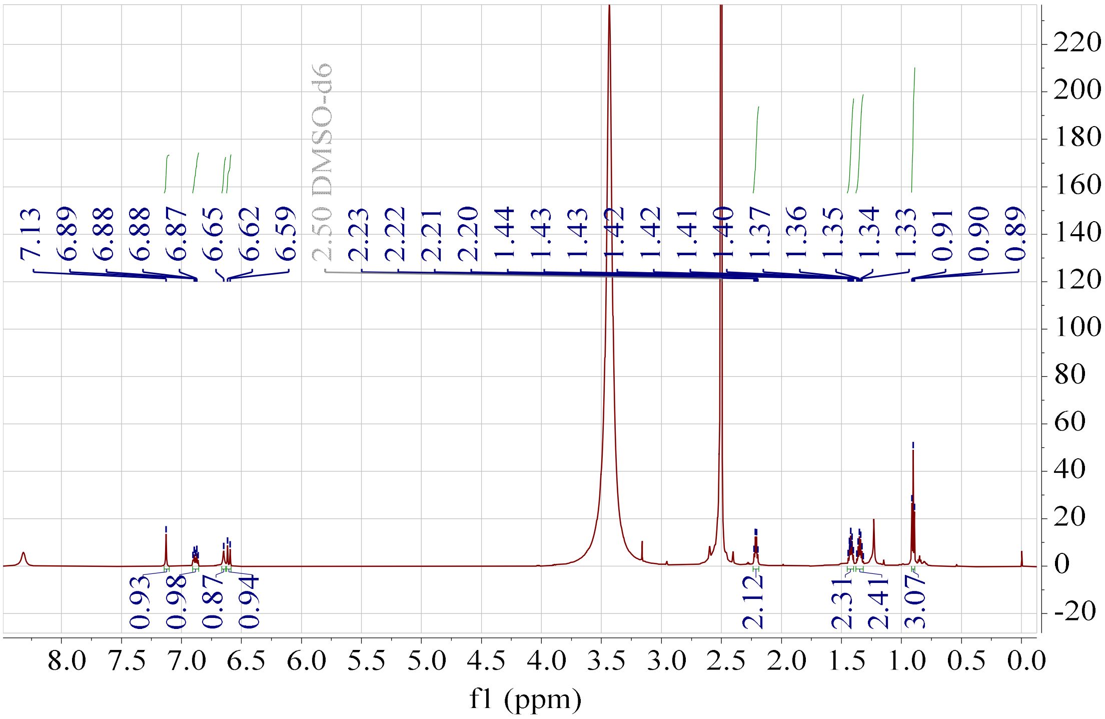

Figure S10. The ^{1}H NMR spectrum of **2** in DMSO-d_6.

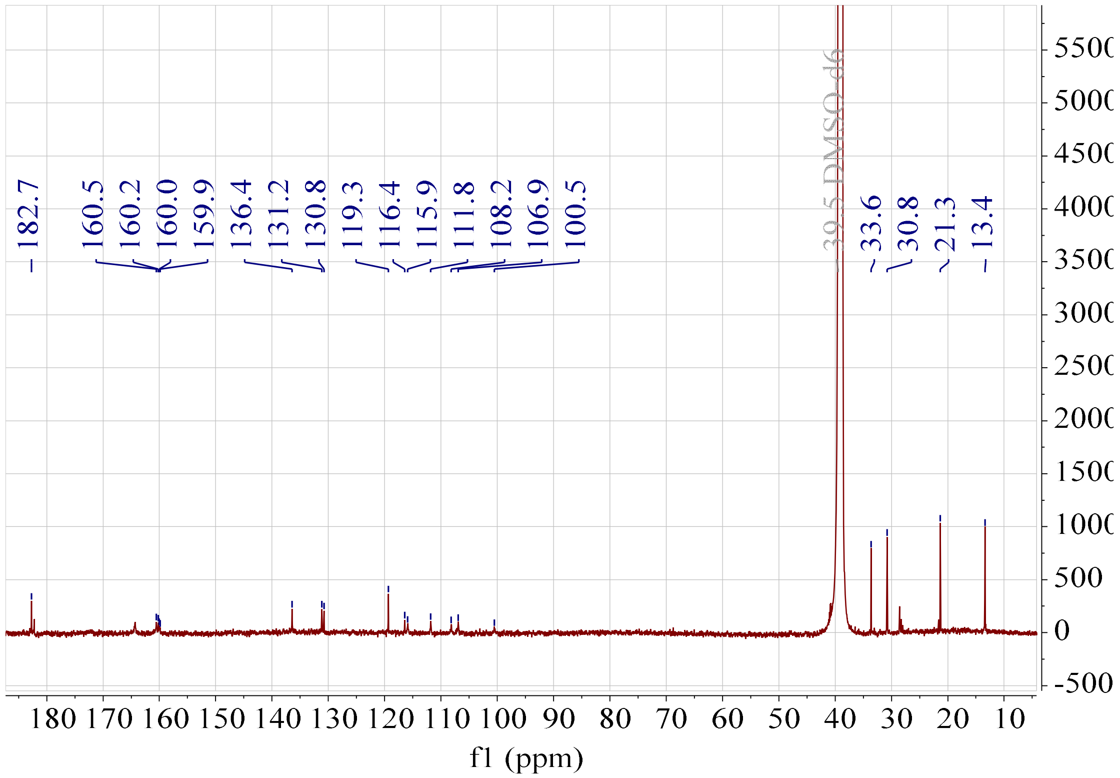

Figure S11. The ^{13}C NMR spectrum of **2** in DMSO-*d*$_6$.

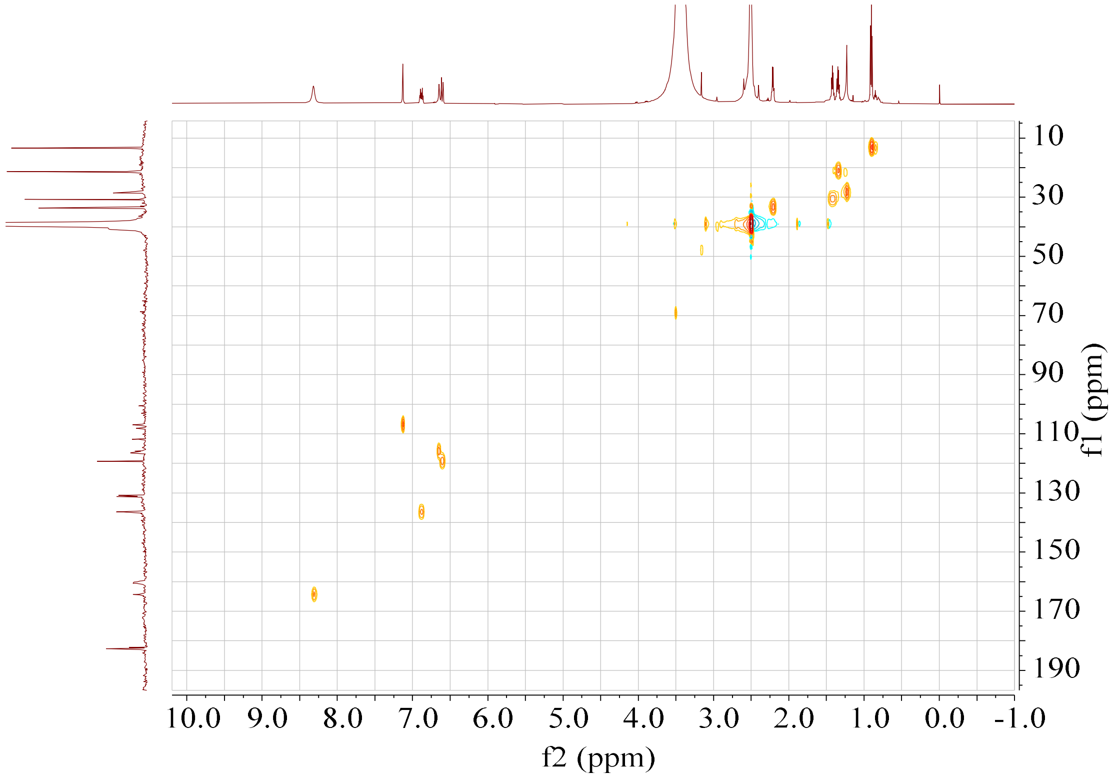

Figure S12. The HSQC spectrum of **2** in DMSO-*d*$_6$.

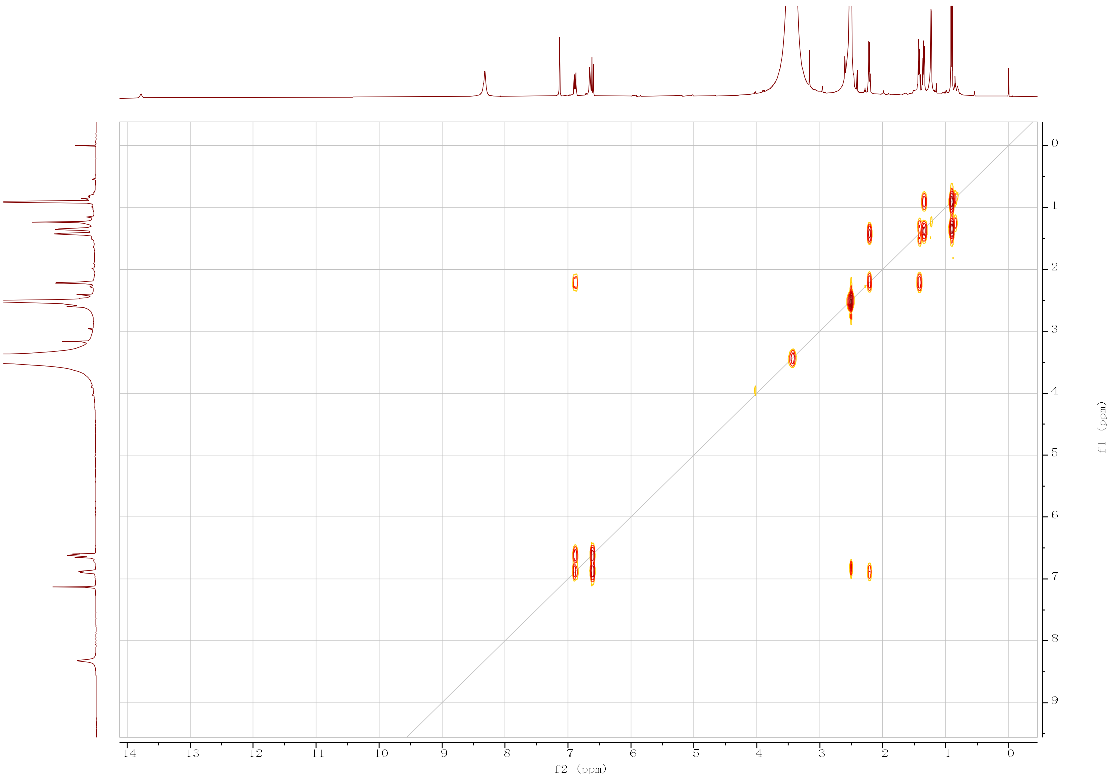

Figure S13. The ^{1}H-^{1}H COSY spectrum of **2** in DMSO-*d*6.

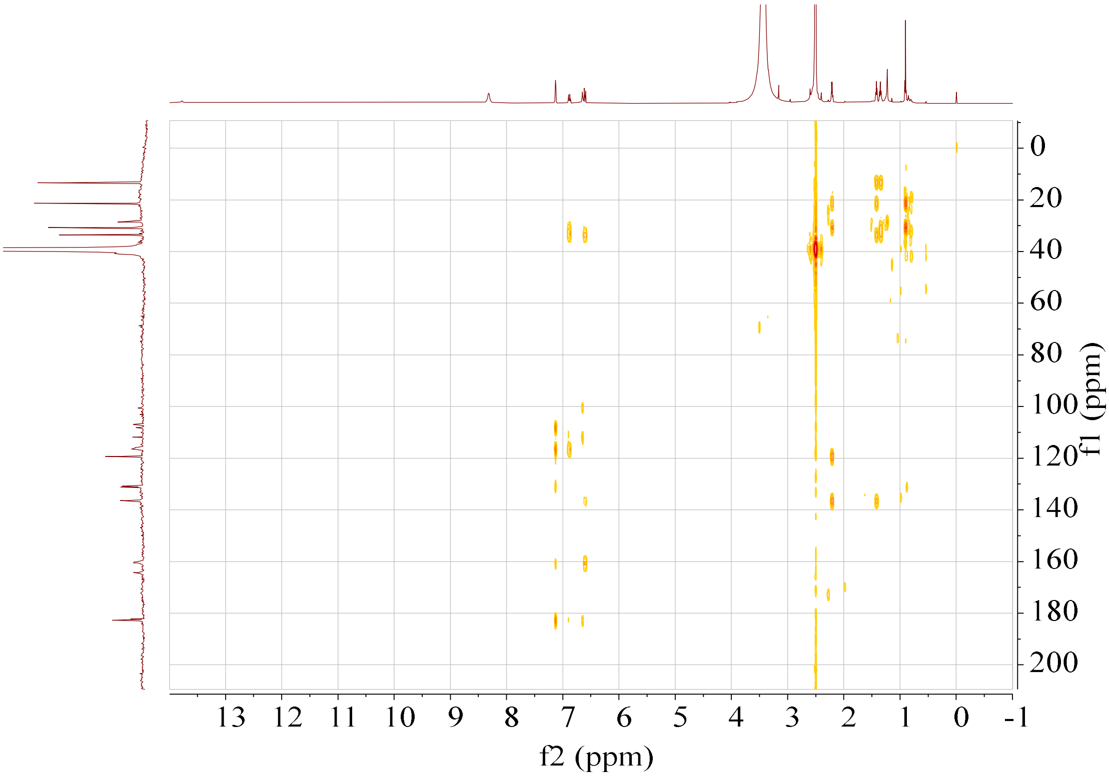

Figure S14. The HMBC spectrum of **2** in DMSO-*d*6.

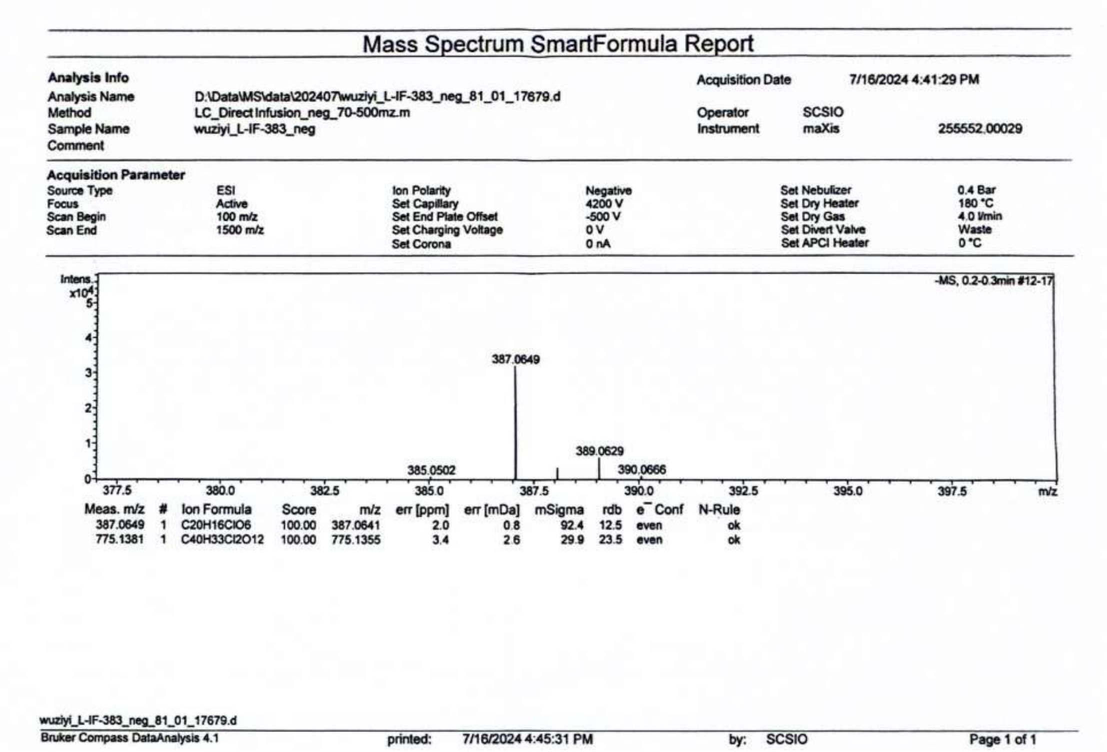

Mass Spectrum SmartFormula Report

**Analysis Info**		Acquisition Date	7/16/2024 4:41:29 PM
Analysis Name	D:\Data\MS\data\202407\wuziyi_L-IF-383_neg_81_01_17679.d		
Method	LC_Direct Infusion_neg_70-500mz.m	Operator	SCSIO
Sample Name	wuziyi_L-IF-383_neg	Instrument	maXis 255552.00029
Comment			

Acquisition Parameter

Source Type	ESI	Ion Polarity	Negative	Set Nebulizer	0.4 Bar
Focus	Active	Set Capillary	4200 V	Set Dry Heater	180 °C
Scan Begin	100 m/z	Set End Plate Offset	-500 V	Set Dry Gas	4.0 l/min
Scan End	1500 m/z	Set Charging Voltage	0 V	Set Divert Valve	Waste
		Set Corona	0 nA	Set APCI Heater	0 °C

Meas. m/z	#	Ion Formula	Score	m/z	err [ppm]	err [mDa]	mSigma	rdb	e⁻ Conf	N-Rule
387.0649	1	C20H16ClO6	100.00	387.0641	2.0	0.8	92.4	12.5	even	ok
775.1381	1	C40H33Cl2O12	100.00	775.1355	3.4	2.6	29.9	23.5	even	ok

wuziyi_L-IF-383_neg_81_01_17679.d
Bruker Compass DataAnalysis 4.1 printed: 7/16/2024 4:45:31 PM by: SCSIO Page 1 of 1

Figure S15. The HRESIMS spectrum of **2**.

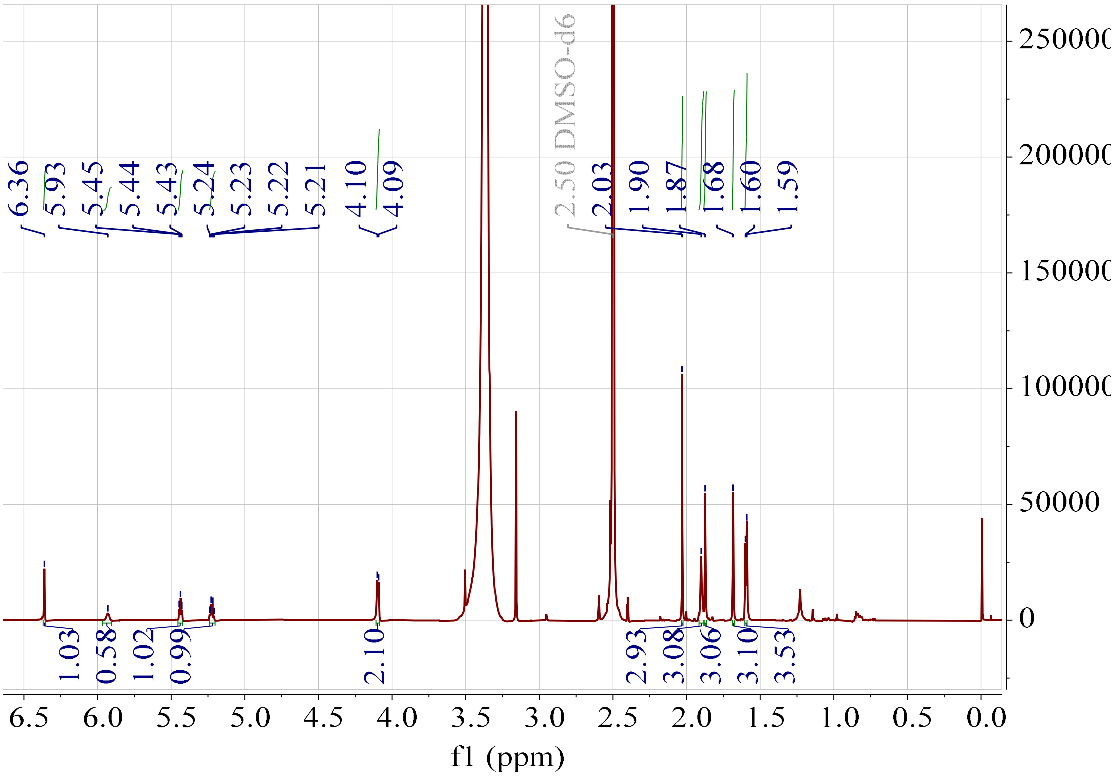

Figure S16. The ^{1}H NMR spectrum of **3** in DMSO-d_6.

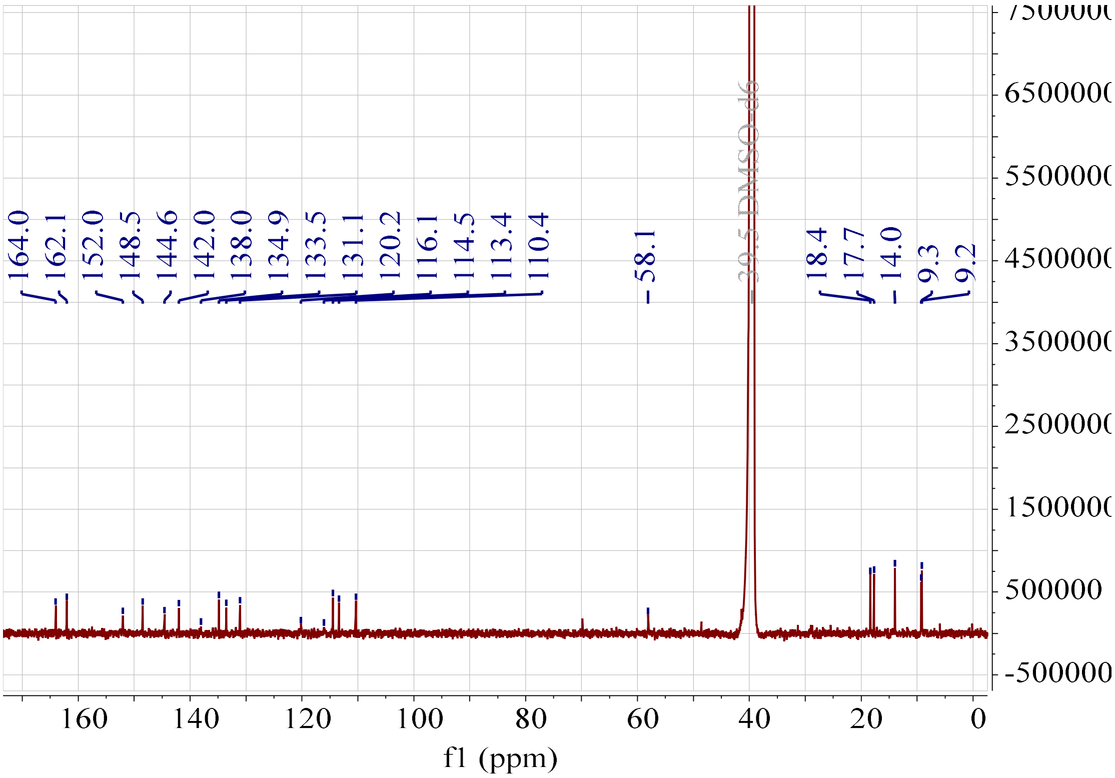

Figure S17. The ^{13}C NMR spectrum of **3** in DMSO-*d*$_6$.

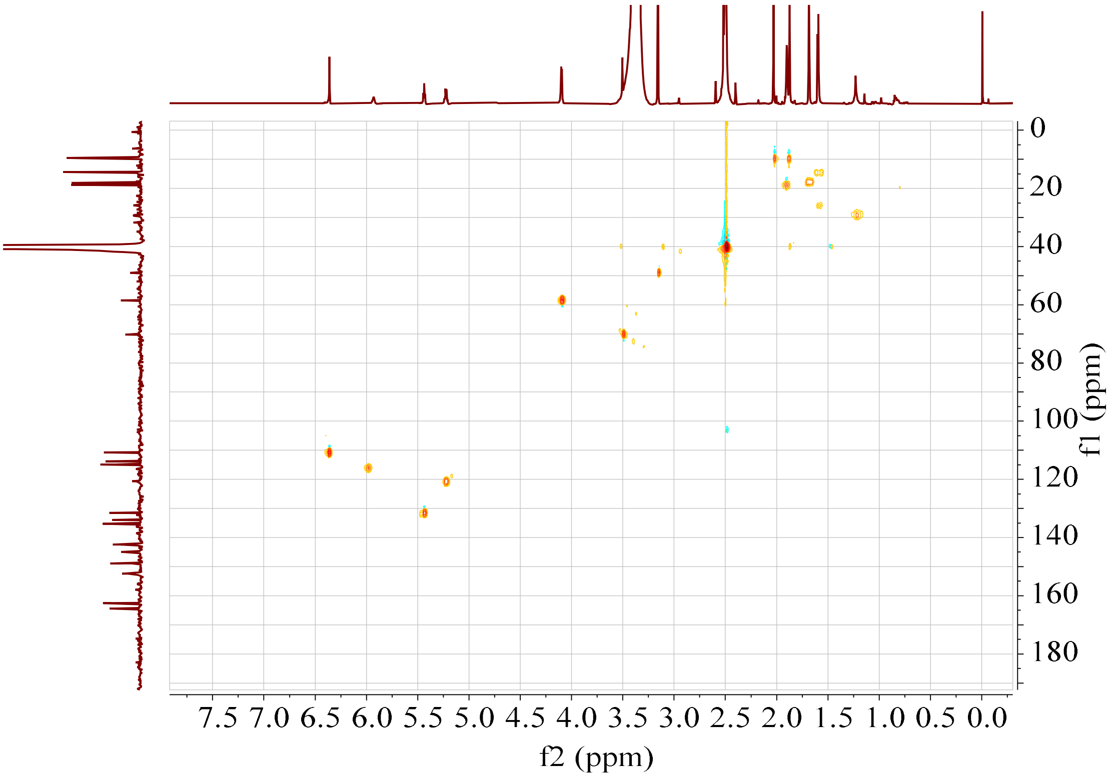

Figure S18. The HSQC spectrum of **3** in DMSO-*d*$_6$.

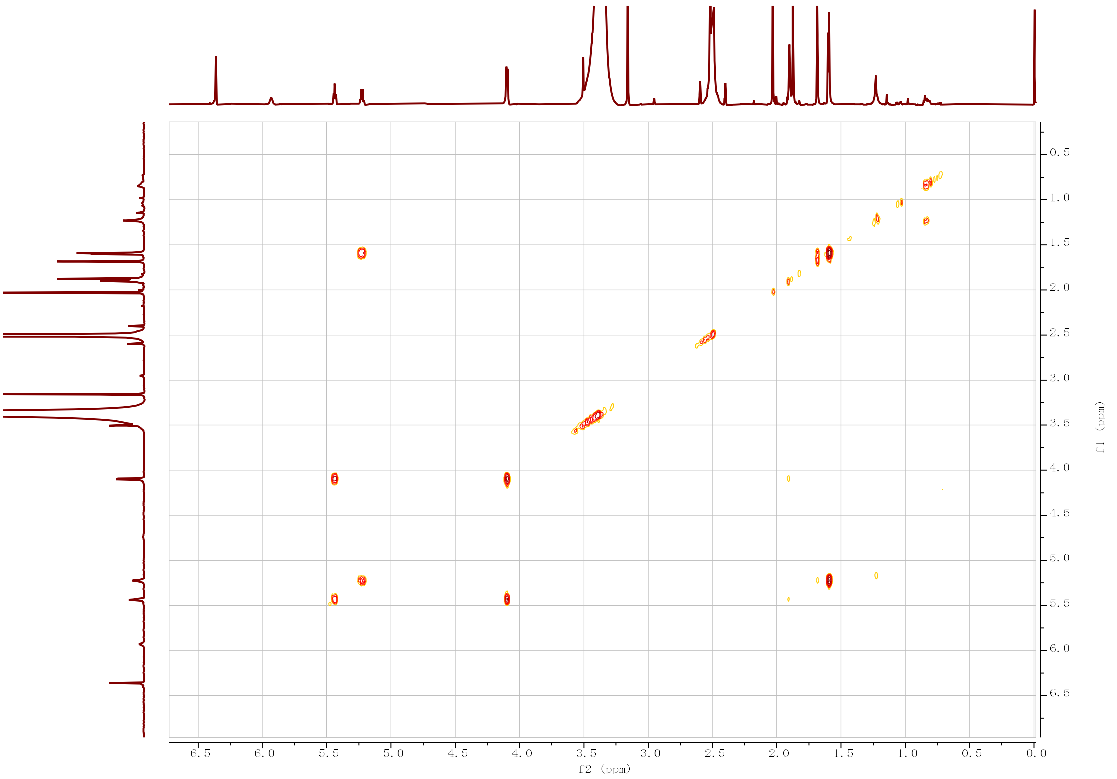

Figure S19. The ^1H-^1H COSY spectrum of **3** in DMSO-d_6.

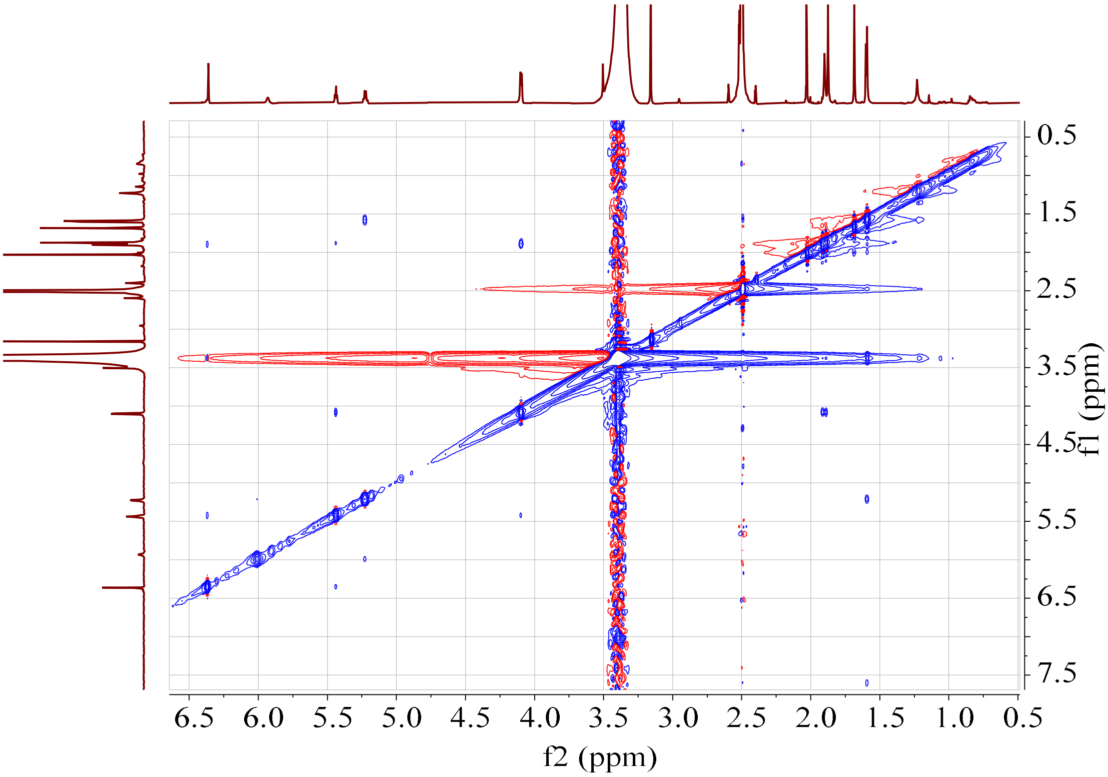

Figure S20. The NOESY spectrum of **3** in DMSO-d_6.

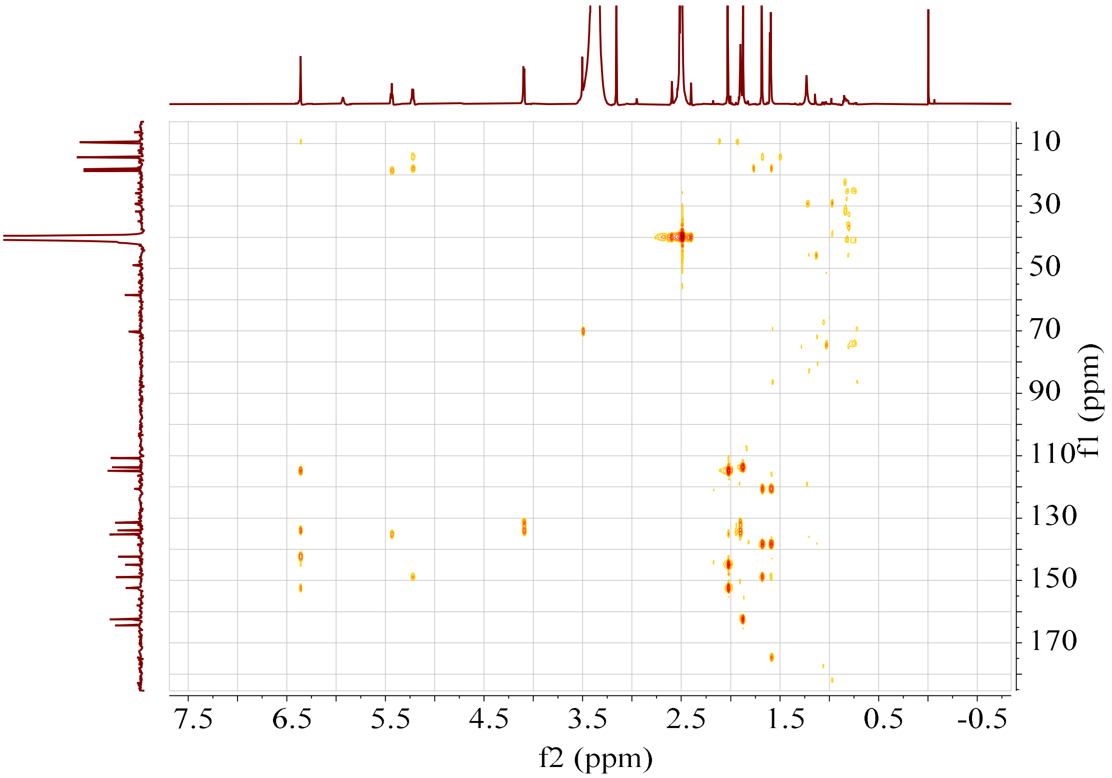

Figure S21. The HMBC spectrum of **3** in DMSO-d_6.

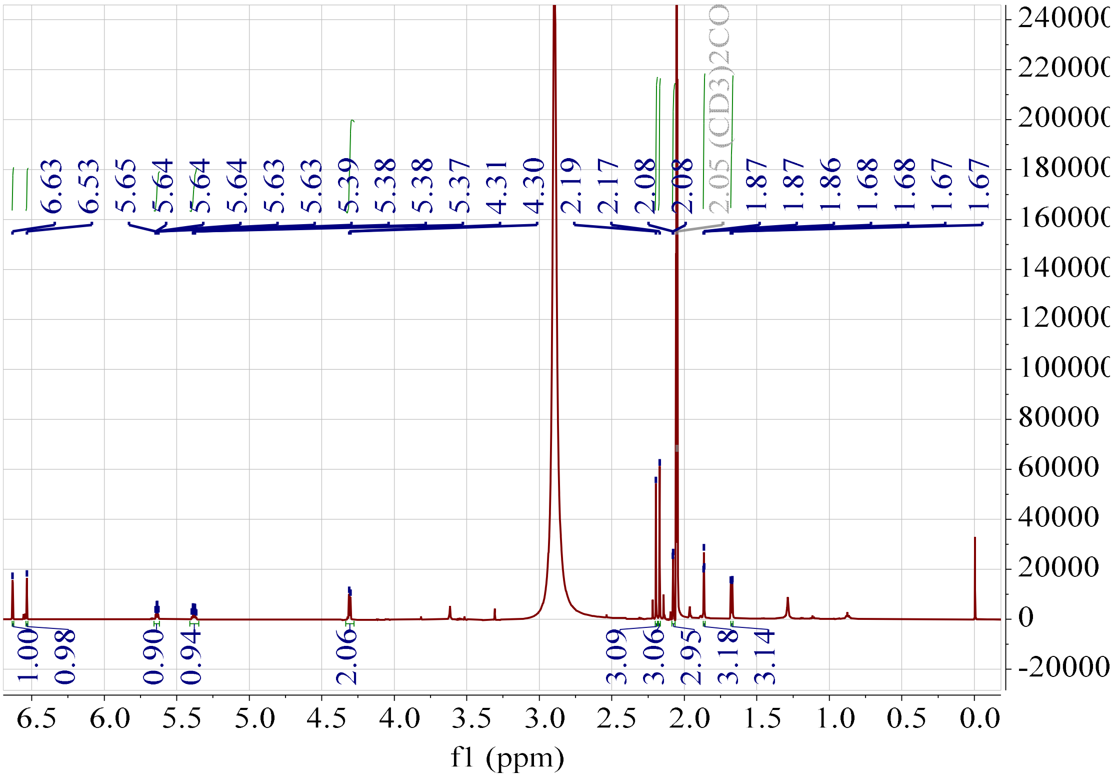

Figure S22. The ^1H NMR spectrum of **3** in acetone-d_6.

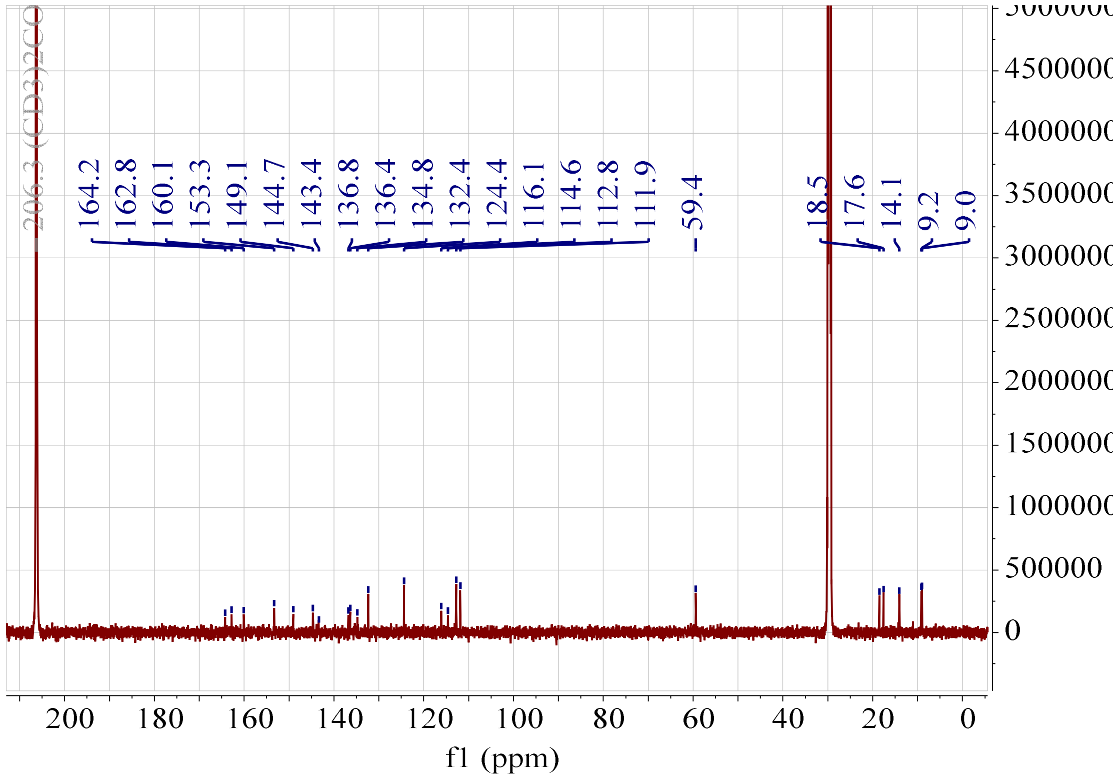

Figure S23. The ^{13}C NMR spectrum of **3** in acetone-*d*6.

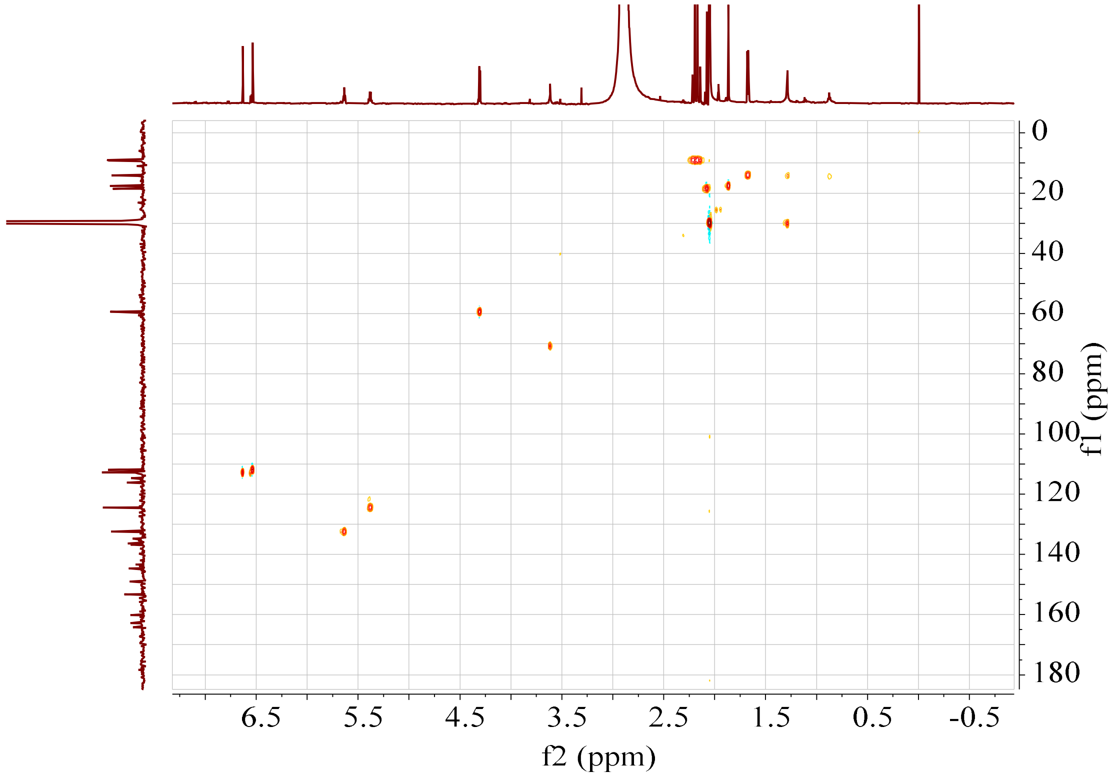

Figure S24. The HSQC spectrum of **3** in acetone-*d*6.

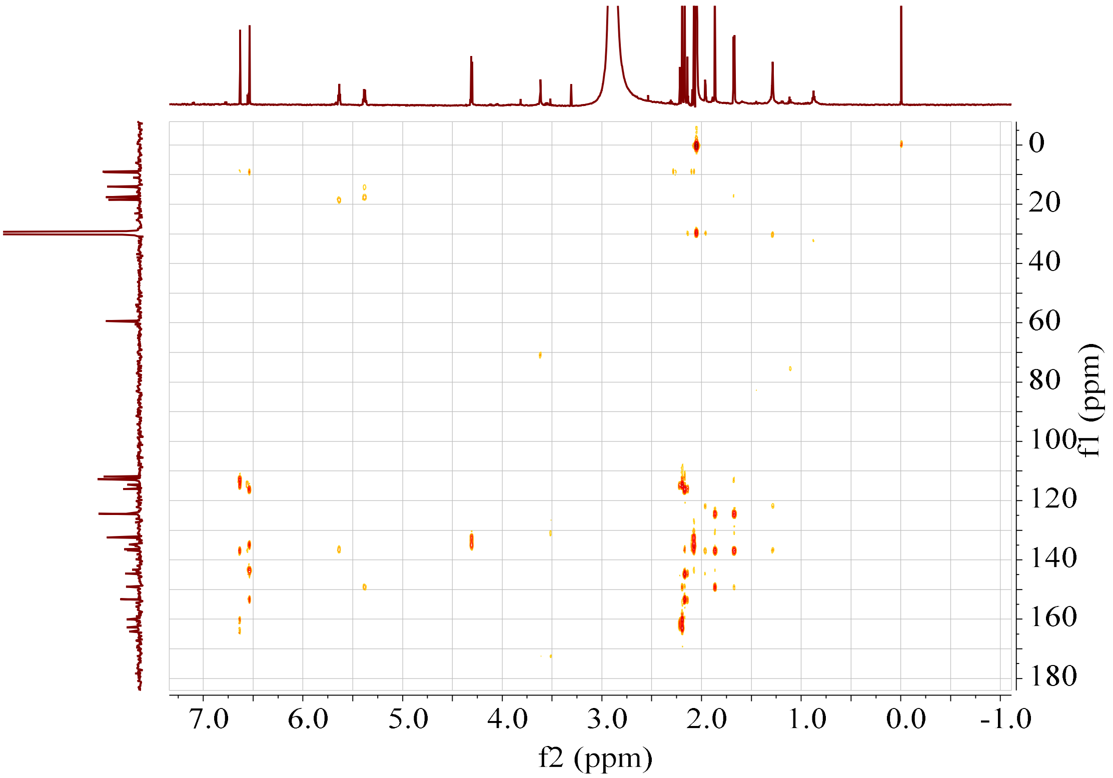

Figure S25. The HMBC spectrum of **3** in acetone-d_6.

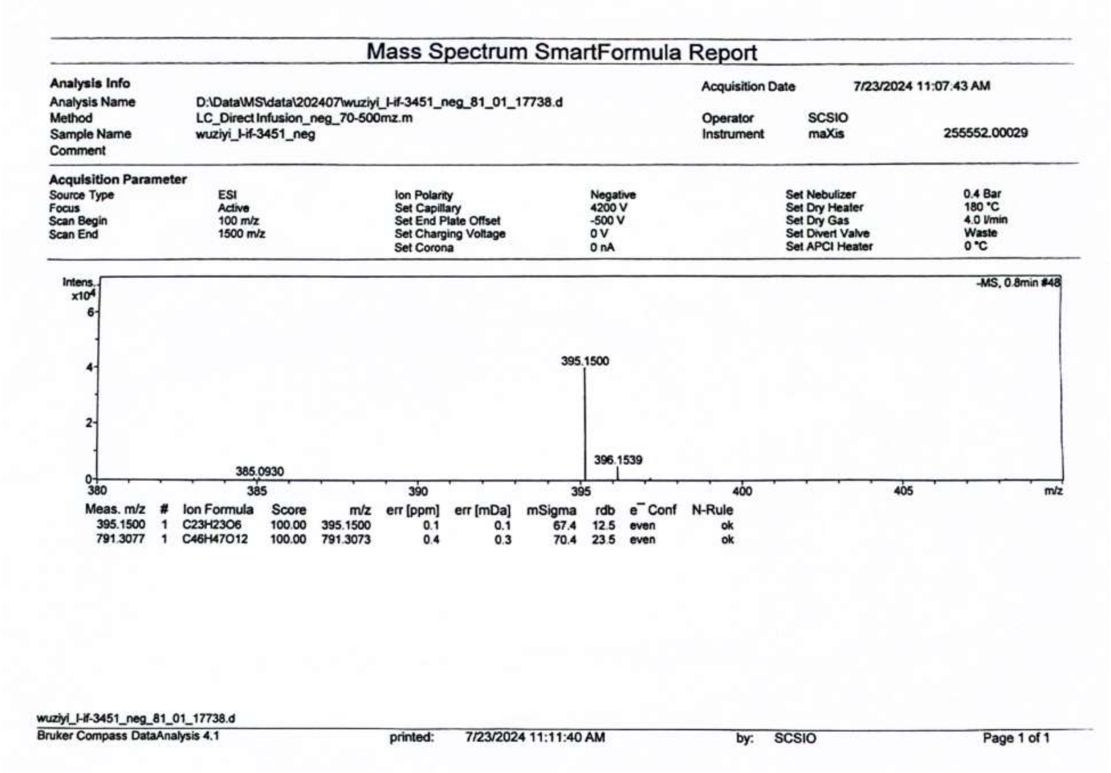

Mass Spectrum SmartFormula Report

Analysis Info		Acquisition Date	7/23/2024 11:07:43 AM	
Analysis Name	D:\Data\MS\data\202407\wuziyi_l-if-3451_neg_81_01_17738.d			
Method	LC_Direct Infusion_neg_70-500mz.m	Operator	SCSIO	
Sample Name	wuziyi_l-if-3451_neg	Instrument	maXis	255552.00029
Comment				

Acquisition Parameter					
Source Type	ESI	Ion Polarity	Negative	Set Nebulizer	0.4 Bar
Focus	Active	Set Capillary	4200 V	Set Dry Heater	180 °C
Scan Begin	100 m/z	Set End Plate Offset	-500 V	Set Dry Gas	4.0 l/min
Scan End	1500 m/z	Set Charging Voltage	0 V	Set Divert Valve	Waste
		Set Corona	0 nA	Set APCI Heater	0 °C

Meas. m/z	#	Ion Formula	Score	m/z	err [ppm]	err [mDa]	mSigma	rdb	e⁻ Conf	N-Rule
395.1500	1	C23H23O6	100.00	395.1500	0.1	0.1	67.4	12.5	even	ok
791.3077	1	C46H47O12	100.00	791.3073	0.4	0.3	70.4	23.5	even	ok

wuziyi_l-if-3451_neg_81_01_17738.d
Bruker Compass DataAnalysis 4.1 printed: 7/23/2024 11:11:40 AM by: SCSIO Page 1 of 1

Figure S26. The HRESIMS spectrum of **3**.

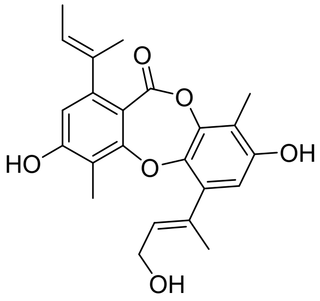

3a (Isomer1)

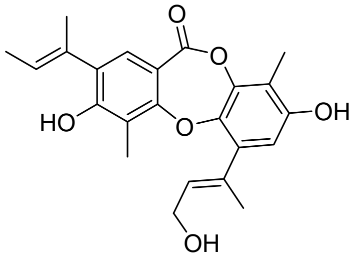

3b (Isomer2)

Figure S27. Structures of the two isomers of **3** used in the DP4+ calculation

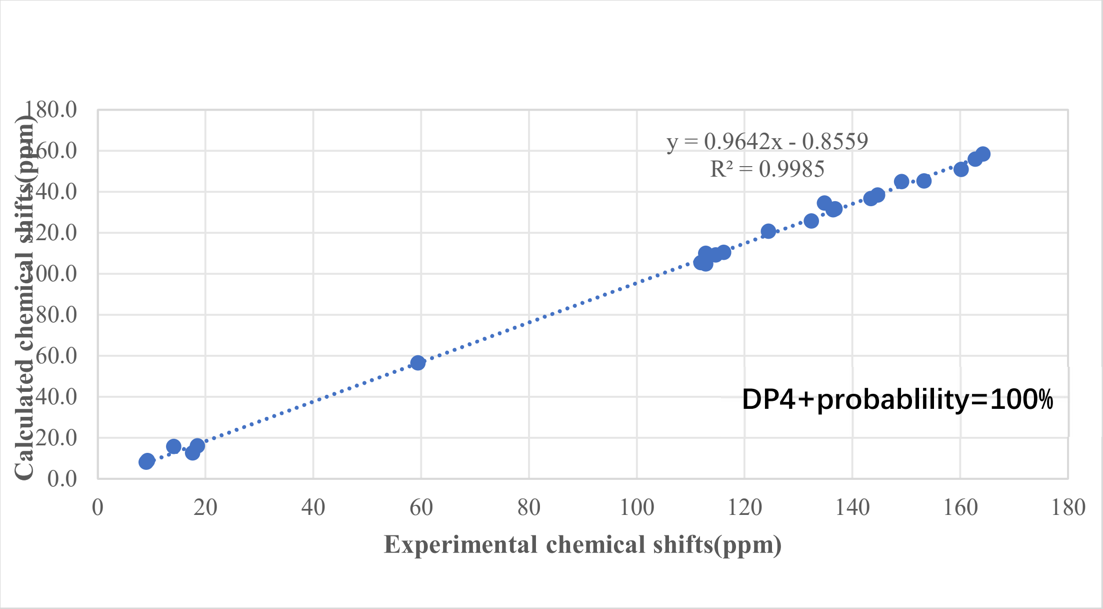

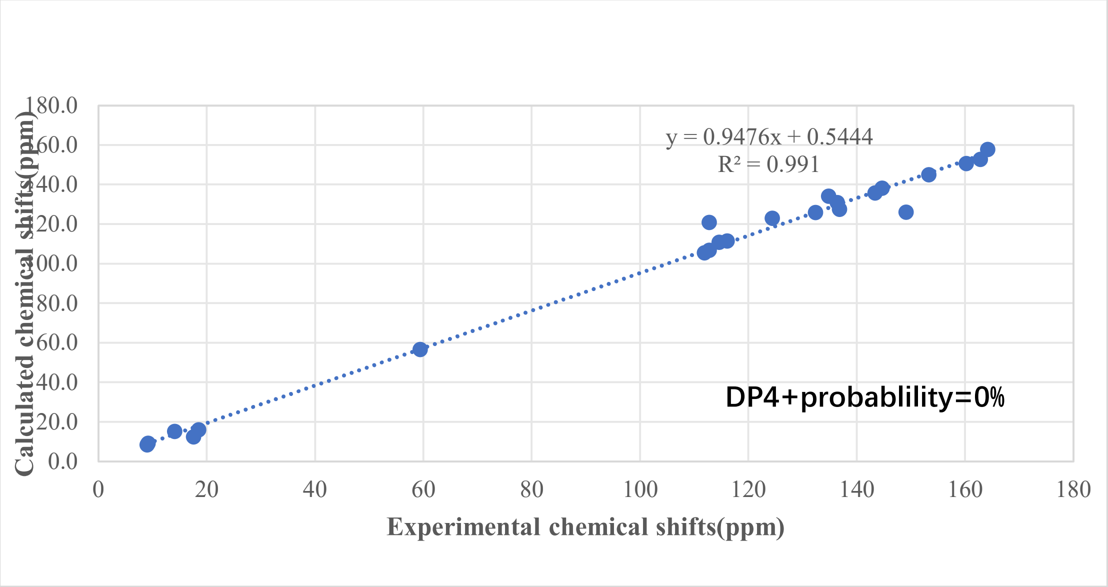

Figure S28. Linear regression analysis of calculated ^{13}C NMR shifts of **3a** (up) and **3b** (down) against the experimental shifts of **3**

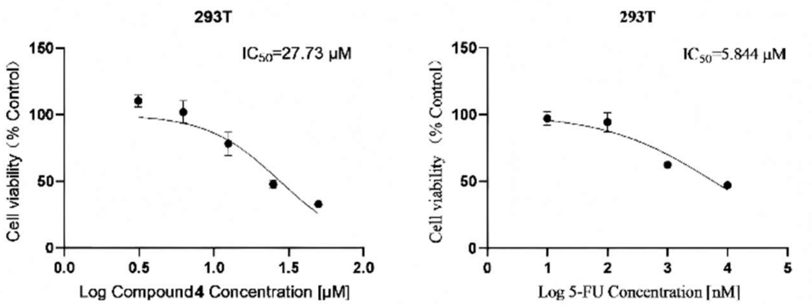

Figure S29. Preliminary cytotoxicity evaluation of compound **4** and 5-FU in 293T cells. Dose–response curves of compound 4 and 5-FU in 293T cells. Cell viability was measured after treatment with increasing concentrations of compound **4** or 5-FU, and IC_{50} values were calculated. Data are presented as mean ± SD (n=3).

Table S1. Energies of dominative conformers of **3a** and **3b** at B3LYP/6-311G(d,p) (acetone)

Conformer	Configuration	E(Hartree)	Energy (kcal/mol)	Population(%)
**3a**-1	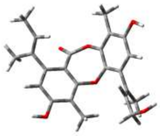	-2.138523668	-1341.94391787	42.75
**3a**-2	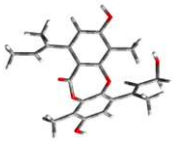	-2.138523595	-1341.94387181	40.71
**3a**-3	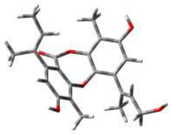	-2.138522239	-1341.94302104	16.54

**3b**-1	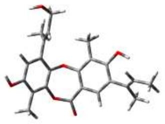	-2.13853557	-1341.95138599	22.10
**3b**-2	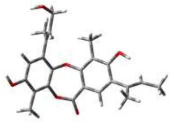	-2.138535456	-1341.95131484	20.49
**3b**-3	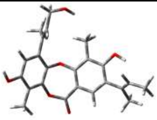	-2.138535766	-1341.95150940	25.18
**3b**-4	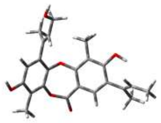	-2.138532654	-1341.94955667	3.18
**3b**-5	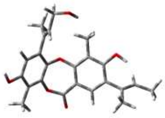	-2.13853582	-1341.95154327	26.10
**3b**-6	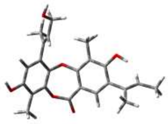	-2.138532537	-1341.94948333	2.95

Table S2. DP4+ analysis of calculated ^{13}C NMR data of compound **3a** (Isomer1) and **3b** (Isomer2)

Functional		Solvent?		Basis Set		Type of Data	
mPW1PW91		PCM		6-31G(d,p)		Unscaled Shifts	
		DP4+	100.00%	0.00%	-	-	-
Nuclei	sp2?	xperimenta	Isomer 1	Isomer 2	Isomer 3	Isomer 4	Isomer 5
C	x	149.1	145.0	126.2			
C	x	112.8	104.8	120.9			
C	x	160.2	150.9	150.8			
C	x	114.6	109.1	110.9			
C	x	162.8	155.8	152.8			
C	x	143.4	136.7	135.8			
C	x	136.4	131.2	131.0			
C	x	111.9	105.4	105.6			
C	x	153.3	145.2	145.1			
C	x	116.1	110.4	111.5			
C	x	144.7	138.3	138.2			
C	x	164.2	158.30	157.90			
C	x	112.8	109.90	106.90			
C		9.2	8.90	9.40			
C		9.0	8.10	8.60			
C	x	134.8	134.50	134.20			
C	x	132.4	125.70	125.90			
C		59.4	56.50	56.80			
C		18.5	16.10	16.10			
C	x	136.8	131.60	127.60			
C	x	124.4	120.80	123.10			
C		14.1	15.80	15.30			
C		17.6	12.60	12.60			
